# Structure, computational and biochemical analysis of *Pc*Cel45A endoglucanase from *Phanerochaete chrysosporium* and catalytic mechanisms of GH45 subfamily C members

**DOI:** 10.1038/s41598-018-21798-9

**Published:** 2018-02-27

**Authors:** Andre S. Godoy, Caroline S. Pereira, Marina Paglione Ramia, Rodrigo L. Silveira, Cesar M. Camilo, Marco A. Kadowaki, Lene Lange, Peter K. Busk, Alessandro S. Nascimento, Munir S. Skaf, Igor Polikarpov

**Affiliations:** 1 São Carlos Institute of Physics, University of São Paulo, São Carlos 13566-590 São Paulo, Brazil; 2 Institute of Chemistry, University of Campinas, Campinas, 13084-862 São Paulo, Brazil; 3 Centro de Tecnologia Canavieira, Fazenda Santo Antonio, PO Box 162, 13400-970 Piracicaba, São Paulo Brazil; 4 Department of Chemical and Biochemical Engineering, Technical University of Denmark, Søltofts Plads, Building 229, 2800 Kgs, Lyngby, Denmark

## Abstract

The glycoside hydrolase family 45 (GH45) of carbohydrate modifying enzymes is mostly comprised of β-1,4-endoglucanases. Significant diversity between the GH45 members has prompted the division of this family into three subfamilies: A, B and C, which may differ in terms of the mechanism, general architecture, substrate binding and cleavage. Here, we use a combination of X-ray crystallography, bioinformatics, enzymatic assays, molecular dynamics simulations and site-directed mutagenesis experiments to characterize the structure, substrate binding and enzymatic specificity of the GH45 subfamily C endoglucanase from *Phanerochaete chrysosporium* (*Pc*Cel45A). We investigated the role played by different residues in the binding of the enzyme to cellulose oligomers of different lengths and examined the structural characteristics and dynamics of *Pc*Cel45A that make subfamily C so dissimilar to other members of the GH45 family. Due to the structural similarity shared between *Pc*Cel45A and domain I of expansins, comparative analysis of their substrate binding was also carried out. Our bioinformatics sequence analyses revealed that the hydrolysis mechanisms in GH45 subfamily C is not restricted to use of the imidic asparagine as a general base in the “Newton’s cradle” catalytic mechanism recently proposed for this subfamily.

## Introduction

As the world demand for alternative sources of energy, chemicals and materials increases, renewable products (including biofuels) derived from plant biomass are emerging as an important field of biotechnological applications^1,2^. However, the recalcitrance and complexity of lignocellulosic biomass have been the main challenges for its enzymatic decomposition^3^, which typically requires the synergistic action of multiple enzymes, including endoglucanases (EGs), exoglucanases and β-glucosidases^4^. Endoglucanases (EC 3.2.1.4) are glycoside hydrolases (GHs)^5^ that predominantly hydrolyze internal β-1,4 cellulose linkages and generate new terminals in the cellulose chains^6^.

The GH45 family is composed mainly of β-1,4-endoglucanases from a number of organisms including plants, animals, bacteria and fungi. Significant diversity between the members of GH45 allowed Igarashi and colleagues to subdivide this family into three subfamilies: A, B and C^6^. The most thoroughly structurally characterized enzymes belong to subfamily A, which is represented by the endoglucanases from *Humicola insolens* (*Hi*EGV, PDB id: 2ENG)^7^ and *Melanocarpus albomyces* (*Ma*Cel45A, PDB id: 1OA9)^8^. The *Hi*EGV acts via an inverting mechanism of reaction^7,9^, utilizing Asp121 and Asp10 as the proton donor and the catalytic base, respectively. On the other hand, *Pc*Cel45A, a member of subfamily C, lacks one of the two catalytic residues and thus employs a distinct catalytic mechanism different from that of the GH45 subfamily A members. It has been recently proposed that *Pc*Cel45A utilizes an imidic acid form of asparagine residue as general base in a “Newton’s cradle” proton relay catalytic mechanism^10^. Yet the evolutionary and biological significance of this adaptation is still unknown, as is the role of active site amino acids, which recognize and participate in cleavage of the cellulose chain.

While subfamily C is significantly dissimilar to canonical GH45 subfamily A enzymes, it is quite similar to the domain I of expansins^6^. Described over 20 years ago^11^, the expansins are plant extracellular proteins that are responsible for plant cell wall expansion through turgor-driven extension for which the mechanism, however, remains unknown^12,13^. Here, we used a combination of structural studies, enzymatic assays and molecular dynamics simulations combined with site-directed mutagenesis experiments and bioinformatics analyses to better understand the mechanisms of substrate recognition and catalytic activity in GH45 subfamily C members.

## Results

### Biochemical characteristics of PcCel45A

**Figure 1:**
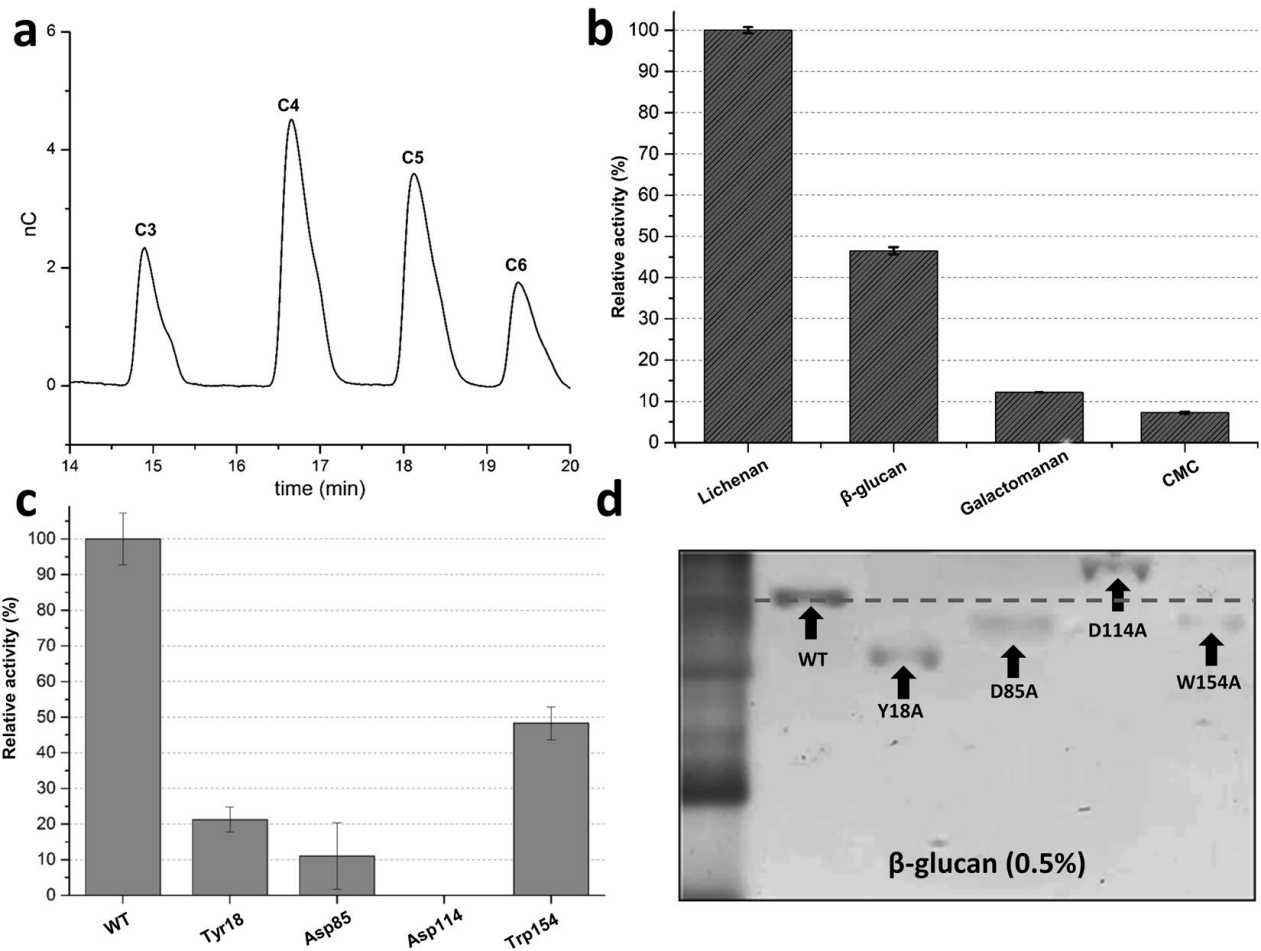
Relative activity of *Pc*Cel45A and its mutants. (**a**) HPAE-PAD chromatogram of soluble oligosaccharide products released from PASC by PcCel45A. (**b**) PcCel45A relative activity against lichenan, β-glucan, galactomannan and CMC. (**c**) WT and mutant relative activities against lichenan. Mutants Y18A, D85A and W154A retained, respectively, 20%, 10% and 48% relative activity compared to the WT, while the mutant D114A lost all activity. (**d**) WT and mutants Y18A, D85A and W154A have faster migration patterns when 0.5% β-glucan is added to the matrix, which suggests weaker interaction with the β-glucan compared to the WT.

*Pc*Cel45A was successfully expressed in the *Aspergillus niger* system and purified (Figure S1). Enzymatic assays using CMC as substrate showed that *Pc*Cel45A has an optimum pH of 4.0, and an optimal temperature of around 70 °C (Figure S2). Enzyme kinetic studies using CMC showed an apparent K_m_ of 2.0 ± 0.6 g/L. *Pc*Cel45A has higher activity against lichenan and β-glucan than toward galactomannan and CMC (Fig. 1). The preference of *Pc*Cel45A for substrates composed mainly of β-1,3/1,4-glucan, has been previously reported^6^. Such specificity differs strikingly from *Hi*EGV which shows clear specificity toward cellulose, CMC, but not lichenan, 1,4-β-D-mannan or xylan^7^. No significant enzymatic activity was detected against Avicel, arabinoxylan and xyloglucan. When the reaction products generated by *Pc*Cel45A from PASC hydrolysis were analyzed by both TLC and HPAEC techniques, we observed the formation of C3–C7 products, but did not detect formation of glucose or cellobiose (Figure S3 and Fig. 1, respectively), which is in line with previous results obtained by Nakamura and coworkers^10^.

### Structure of PcCel45A

*Pc*Cel45A crystal structure refined to 1.5 Å resolution revealed a wide active site groove with approximately 50 Å in length, 13 Å in depth and 12 Å in width at the surface of the enzyme (Figure S4). In the GH45 subfamily A structure^7,9^, this groove contains the putative catalytic residues, including two catalytic aspartate residues. The structure of the *Pc*Cel45A-cellobiose complex revealed a single cellobiose molecule within the active-site groove (Figure S5). The structure of apo *Pc*Cel45A determined here has a RMSD of 0.11 Å (computed for all 923 atoms) when compared to the previously solved apo- and cellopentaose bound structures of *Pc*Cel45A (PDB ids 3X2L and 3X2M^10^).

The residues Asp114, Met17, Gly131, Tyr67 and Asn92 in *Pc*Cel45A X-ray structure form direct hydrogen bonds with the cellobiose. At the +2 position, the amide nitrogen of Met17 (N) and the carbonyl oxygen of Gly131 (O) interact with O6’ of cellobiose, and possibly stabilize the ligand in the active site. Furthermore, the O2’ atom forms a hydrogen bond with the hydroxyl of the Tyr67 phenyl ring (2.9 Å). At the +1 position, the O2’ hydroxyl forms a hydrogen bond with the carbonyl oxygen of Met17.

The Asn92 OD1 forms hydrogen bonds with O4 and O6 (2.7 and 3.3 Å) at the +1 position. Another carboxyl group (OD2) of the catalytic Asp114 side chain interacts with the non-reducing end of cellobiose (O4) at the +1 position (2.7 Å). Cellobiose is also stabilized by weak interactions with Thr16, Tyr18 and Phe95, and by hydrogen bond interactions mediated by water molecules with the residues Ser19 and Glu94. The comparison between apo-*Pc*Cel45A structure and its complex with cellobiose reveals that the ligand binding induces a small conformational change in B1 and B5 strands, where the residues Met17 and Asp114 are respectively located. Comparison of the active sites of *Pc*Cel45A, *Hi*EGV and *Ma*Cel45A complexed with the ligands showed that cellobiose occupies a similar position when related to Asp114 and the proton donor. In all cases cellobiose is located at the +1 and +2 subsites of the active sites.

### Comparison between PcCel45A and other representative GH45 members

**Figure 2:**
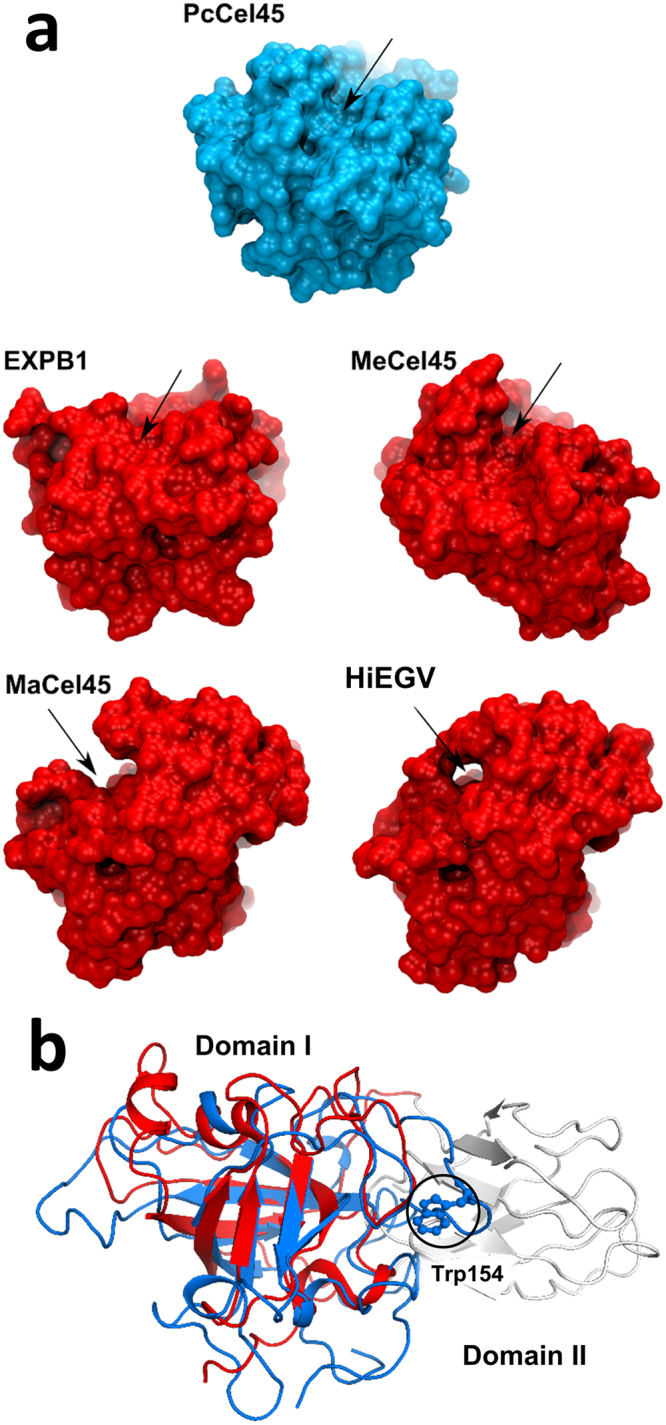
Comparison between *Pc*Cel45A and other members of family 45 and homologues. (**a**) *Pc*Cel45A (blue) and EXPB1 (red) active sites (indicated by arrows) have much shorter loops than homologues *Ma*Cel45A (red) and *Hi*EGV (red) structures. (**b**) Superposition of PcCel45A with domain I of expansins. *Pc*Cel45A (green) is notably similar to the domain I of EXPB1 (red). In *Pc*Cel45A, Trp154 (highlighted with black circle) is in a similar position in the EXPB1 domain 2 (grey).

Despite the same fold (double psi-β-barrel) and the homologous sequence, the *Pc*Cel45A structure is significantly different from *Hi*EGV and *Ma*Cel45A^7,8^. The most canonical EGs from GH45 family, such as *Hi*EGV and *Ma*Cel45, have a flattened sphere shape, with the active site groove passing through the sphere and being surrounded by loops (Fig. 2)^7–9^. In contrast, *Pc*Cel45A has the shape of an anchor and its catalytic groove runs across the surface of the structure which contains most of the catalytic residues, including the conserved Asp114, but with more discrete loops (Fig. 2 and Figure S4).

In *Hi*EGV, the Asp10 is located in the loop 1, in an anti-parallel position to the conserved Asp121 and separated by 11.3 Å, in a typical orientation for catalysis with inversion of the anomeric configuration. The Asp10 has been characterized as a proton acceptor in the GH45 subfamily A enzymes^7,9^, but the corresponding aspartate residue does not exist in the *Pc*Cel45A structure. Instead, *Pc*Cel45A has Asp85 located in the groove (B4” strand), at a distance of about 8.0 Å from Asp114 (Figure S5). The distances between Asp121-Asp10 (in *Hi*EGV) and Asp114-Asp85 (in *Pc*Cel45A) are similar, but the parallel positioning of Asp85 and Asp114 residues does not favor the classic inversion mechanism. Consequently, it has been proposed that Asn92 acts in its imidic acid form and plays a role as a general base of the reaction^10^.

The *Mytilus edulis* EG (*Me*Cel45A, PDB id 1WC2, not published), the only member of GH45 subfamily B with a publicly available structure, is more similar to *Pc*Cel45A (with a RMSD of 0.6 Å for 314 atoms; Figure S4). This enzyme has conserved catalytic base and acid residues (Asp24 and Asp132) in the positions that are similar to those observed in *Hi*EGV structure^9,14^. Yet, very little information is available about activity and catalytic mechanism of this subfamily of enzymes.

### Comparison of PcCel45A with expansins

In line with previous phylogenetic studies^6^, *Pc*Cel45A has greater structural similarities with domain I of expansins than with the canonical members of the GH45 family. A DALI^15^ search for structural homologues of *Pc*Cel45A revealed that *Me*Cel45A, a member of the GH45 subfamily B, is the closest structural homologue of *Pc*Cel45A (Z-score of 13.1 and 28% of amino acid sequence identity) followed by several members of the expansin family, with Z-scores greater than 10 and their sequence identities ranging from 16 to 21%. For sake of comparison, DALI ranks the structural similarity between *Pc*Cel45A and HiEGV (PDB id 2ENG) with a Z-score of 5.3, despite 24% sequence identity between these two enzymes.

A structural comparison of the domain I from the expansin subfamily group-1 grass pollen allergens (EXPB1, PDB id: 2HCZ)^16^ results in an RMSD value of 0.87 Å (for 138 Cα atoms) and also 15% of sequence identity with *Pc*Cel45A (Fig. 2). The conserved motif termed HFD (histidine, phenylalanine, aspartic acid)^17^ is part of the conserved GH45 active site, and is also present in *Pc*Cel45A. The HFD motif corresponds to the residues 112-114, maps onto the strand β5 and contains the catalytic aspartate residue Asp114 in the *Pc*Cel45A structure. In EXPB1, the HFD motif corresponds to the residues 105–107 and also maps onto the strand β5.

Notably, the corresponding second catalytic residue Asp10 is absent in both *Pc*Cel45A and EXPB1 structures. However, the Asp95 residue in EXPB1 is strongly conserved in the expansins subfamily and could be related to the residual enzymatic activity observed in these proteins^16,17^. *Pc*Cel45A contains an aspartic residue in the equivalent position (Asp85) which might play a role in the catalytic activity of this enzyme.

### Comparison of the enzymatic activity of PcCel45A mutants

The Asp114 is largely conserved and its importance for catalysis has already been clearly established^6,10^. The triad Asp114, Asp85 and Tyr18 is conserved both in the expansins and the active sites of lytic transglycosylases^16,18^. To check their role in catalysis, we mutated each one of these three amino acid residues into Ala using site-directed mutagenesis. Moreover, Trp154 was also mutated due to its position at the catalytic site and its spatial correspondence with the tryptophan from the domain II of expansins (Figure S5). Similarly, mutation of Tyr67 was also introduced but resulted in insoluble protein (data not shown). The specific activities of all mutants were compared with the specific activity of the native protein on β-glucan. Not surprisingly, D114A lost all its enzymatic activity. The activity of D85A was greatly reduced to 10% of the WT reference value, showing that this residue is important but not essential for the catalysis. The Y18A mutant lost approximately 80% of its original activity. The enzymatic activity of W154A was less affected, with 50% of the original catalytic activity preserved (Fig. 1). The migration pattern of mutants in the electrophoretic acrylamide gel prepared with β-glucan indicated that mutants Y18A, D85A and W154A have lower affinity for β-glucan than the WT enzyme, which suggests that these residues may play a role in substrate recognition and stabilization (Fig. 1).

### Binding to oligosaccharides

**Figure 3:**
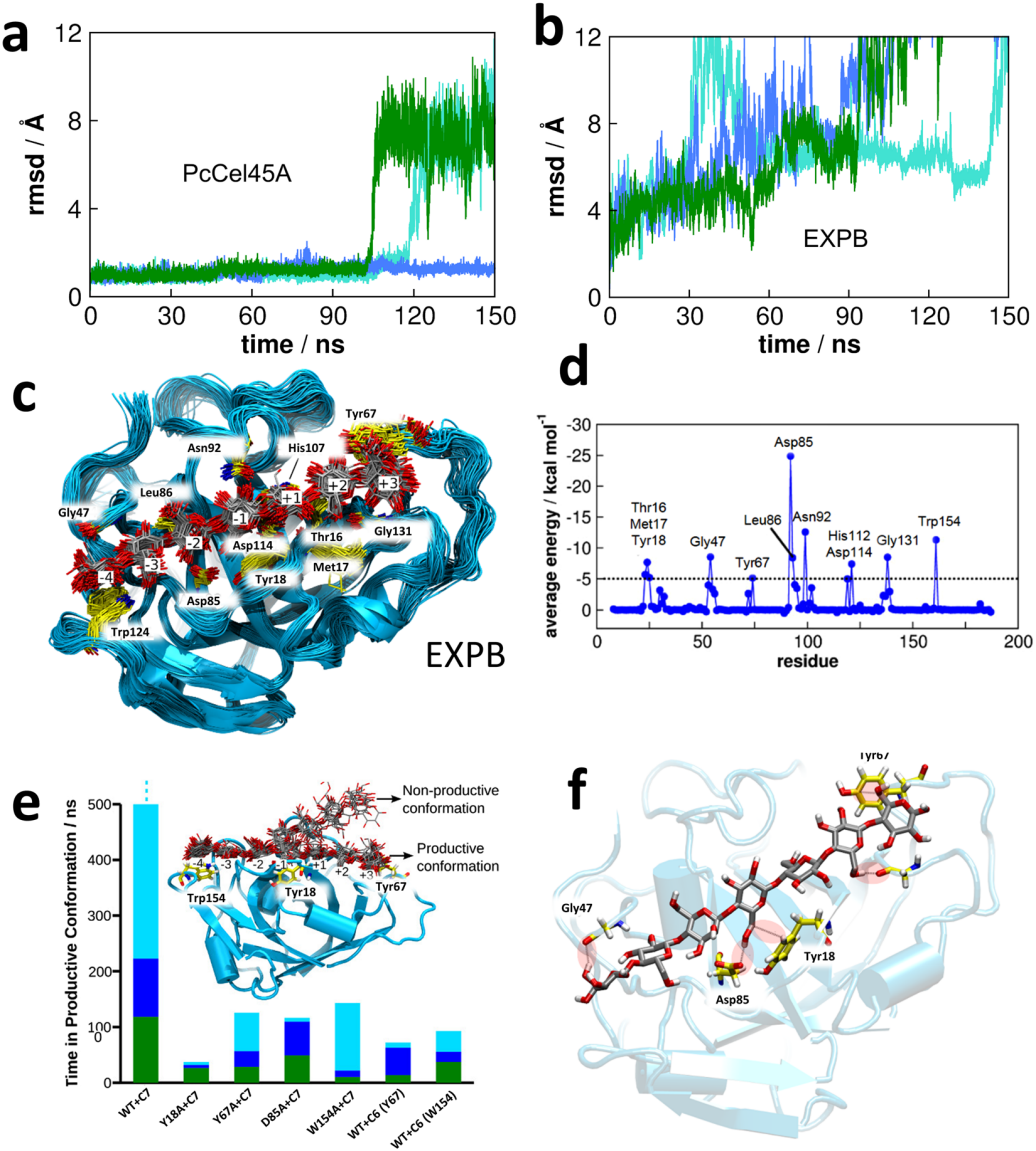
Substrate dynamics of PcCel45A. (**a**) RMSD (root mean squared deviation) of the bound C7 chain from its initial configuration. When the RMSD fluctuates around ~2 Å, the C7 chain is bound in the productive conformation. (**b**) When EXPB1 is bound to C7, RMSD increases to ~8 Å, and the C7 chain assumes a non-productive binding mode. Different colors represent different simulations of the same system. (**c**) Superposed configurations of the *Pc*Cel45A-C7 complex showing the residues that interact most with the substrate. Binding subsites towards the reducing end after the active site are labeled by positive integers; binding subsites towards the non-reducing end after the active site are labeled by negative integers. The active site lies between the −1 and +1 subsites. (**d**) Average interaction energies between the C7 chain and the *Pc*Cel45A. (**e**) Time during which the substrate (C7 and C6) remains in the productive conformation in different variants of *Pc*Cel45A illustrated for different simulations (different colors). The *Pc*Cel45A-C7 complex is the most stable regarding substrate binding. In one of the WT + C7 simulations (cyan), the substrate did not deviate from the initial docking during 500 ns of simulation, and this is indicated by an ellipsis over the cyan bar. Productive and non-productive conformations are illustrated in the inset, showing that the deviation of the productive states happens in the positive subsites. (**f**) Interactions between *Pc*Cel45A and the hydroxymethyl exocyclic groups of the C7 chain.

To further explore enzyme-substrate interactions, we used molecular dynamics (MD) simulations to study the binding mode of cellooligosaccharides to the *Pc*Cel45A. Initially, a model structure of the *Pc*Cel45A-celloheptaose (C7) complex was built as follows: (1) two cellotriose (C3) spanning subsites −4 to −2 and +1 to +3 were taken from the *Humicolas insolens* EGV enzyme^7^ (PDB id: 4ENG) after alignment of the binding site residues with *Pc*Cel45A and (2) an additional glucosyl residue was added to the −1 subsite (details are given in the SMI text). In the simulations, the C7 chain remained tightly bound to the enzyme in a configuration suitable for hydrolysis for hundreds of nanoseconds (Fig. 3), after which the chain started deviating from the productive binding mode, while such chain deviation was not observed in the EXPB1 simulation (Fig. 3).

While the C7 is bound in the productive conformation (Fig. 3c), the RMSD fluctuates around 2 Å, and when the C7 deviates from the productive conformation, the RMSD increases abruptly to 8 Å. Figure 3d shows the average interaction energies between C7 and each residue of *Pc*Cel45A, which demonstrates that the strongest enzyme-substrate interactions are associated with the residues Asp85 (the −2 subsite), Asn92 (the −1 subsite) and Trp154 (the −4 subsite), all of which are in the negative subsites. The residues that interact with the C7 chain with energies of at least −5 kcal.mol^−1^ are shown in Fig. 3c. The C7 chain is stable in most of the subsites and only glucosyl bound to Tyr67 (the +3 subsite) has a higher mobility. This is consistent with the notion that when the cellulose chain assumes a non-productive binding mode, it is only the part of it bound to the positive sites that loses interactions with the enzyme (Fig. 3e).

In MD simulations of xyloheptaose (X7) bound to *Pc*Cel45A, X7 completely dissociated from the enzyme within the first ~5 ns of the simulations (Figure S7), thus revealing a very low affinity between xylan and *Pc*Cel45A. This most likely explains the lack of activity of *Pc*Cel45A against xylan. Xylose differs from glucose by the absence of the hydroxymethyl exocyclic groups. Analysis of the *Pc*Cel45A-C7 complex shows that the exocyclic groups in fact play major roles in the carbohydrate recognition by the enzyme: about a third of the enzyme-substrate interactions come from the exocyclic groups (−100 kcal.mol^−1^ for endocyclic groups and −57 kcal.mol^−1^ for exocyclic groups), and Tyr18, Gly47, Tyr67 and Asp85 are the residues involved in such interactions, contributing, respectively, with −6, −5, −12 and −25 kcal.mol^−1^ for cellohexaose binding (Fig. 3).

### Conformational stability of Asn92 and proton transfer mechanism

**Figure 4:**
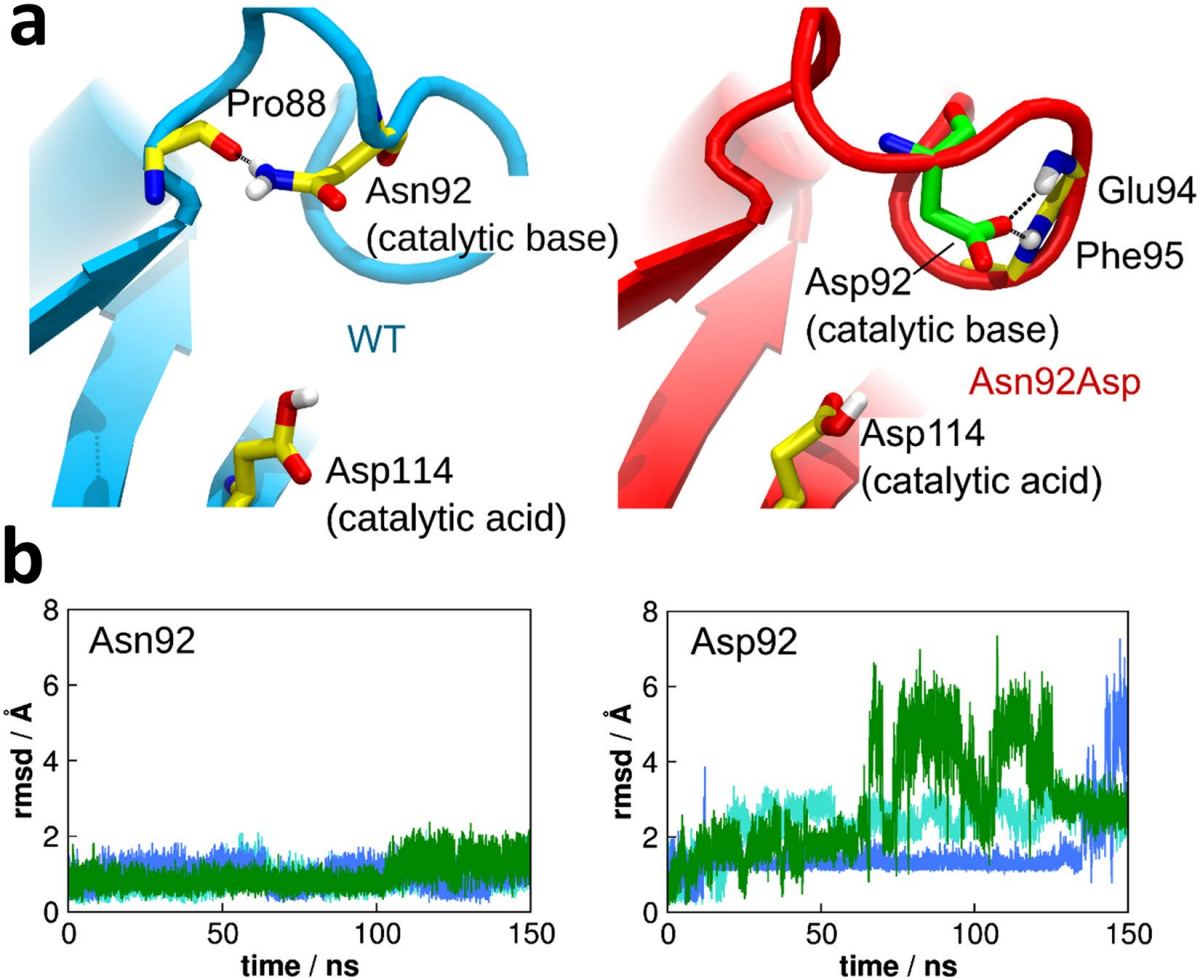
Conformational changes caused by the mutation Asn92Asp. (**a**) Conformation of Pro88 in the WT (cyan) and Asn92Asp (red) variant of *Pc*Cel45A. In the WT enzyme, Asn92 makes a hydrogen bond with the backbone of Pro88. In the Asn92Asp variant, the Asp92 moves away from Pro88 and establishes hydrogen bonds with Glu94 and Phe95. (**b**) RMSD of residues Asn92 and Asp92 showing that the catalytic base is quite stable in the WT *Pc*Cel45A but gains mobility upon the mutation of Asn92 to Asp. Different colors correspond to different independent simulations of the same system.

As has recently been shown by Nakamura *et al*.^10^, the residue Asn92 acts as a general base in the catalytic mechanism proposed for *Pc*Cel45A. Our simulations of *Pc*Cel45A-C7 complex showed that this residue remained stable in a crystallographic-like conformation during the simulation time, and exhibited RMSD from its initial configuration below ~2 Å (Fig. 4). In such a conformation, the NH_2_ group of the Asn92 side chain establishes a hydrogen bond with the carbonyl group of the Pro88 backbone so that the carbonyl group of the Asn92 side chain remains free (Fig. 4). This shows that Asn92 adopts a well-defined conformation, as would be expected for a catalytic residue.

When Asn92 is replaced by an aspartic acid, the enzyme activity drastically decreases^10^, even though Asp92 could in principle also act as a general base. Our simulations of the N92D mutant reveal that, unlike Asn92, residue Asp92 is mobile, reaching RMSD values of up to ~7 Å (Fig. 4). This suggests that the very low activity of the N92D mutant could be associated with the lack of structural stability of the catalytic base with respect to the substrate. The reason for such structural changes is that the carboxylate group of Asp92 cannot form a hydrogen bond with the carbonyl group of Pro88 backbone as a donor, while the amide group of Asn92 can form such a bond. Instead, Asp92 moves away from Pro88 and establishes interactions with the -NH group of the backbone of residues Glu94 and Phe95 as hydrogen bond receptors (Fig. 4). These interactions prevent Asp92 from effectively acting as a catalytic base in the inverting mechanism of *Pc*Cel45 A.

**Figure 5:**
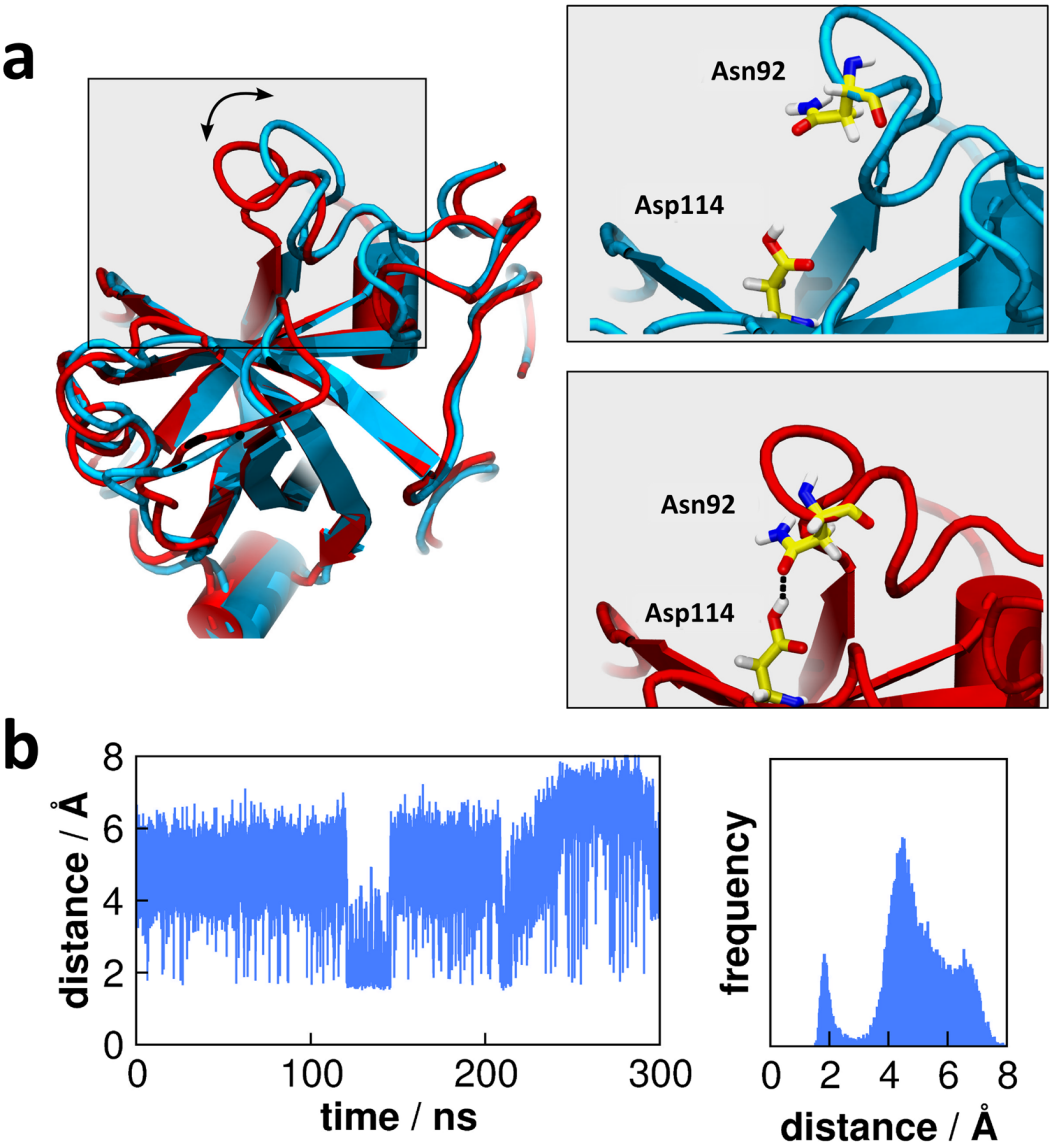
Loop dynamics of *Pc*Cel45A in the absence of substrate. (**a**) (*left*) Two different conformations of the loop that contains the catalytic base Asn92 sampled by MD. (*right*) Amplified view of the Asn92 loop, showing that, in the closed (red) conformation the catalytic residues Asn92 and Asp114 interact with a hydrogen bond. In the open conformation (blue), these residues are in the same position as seen in the crystallographic structure. (**b**) Distance between Asn92 and Asp114 in function of the time (*left*) and represented as a distribution (*right*), showing that such residues approach each other (distance of ~2 Å at around 125 ns and 210 ns). For most of the time, however, the residues remain in the crystallographic position which is suitable for substrate binding. We suggest that proton transfer between the catalytic residues may happen when the loop closes.

Our MD simulations revealed that in the absence of substrate the loop that contains the catalytic residue Asn92 exhibits open-close motions (Fig. 5a). When the loop assumes the closed conformation, the catalytic residues Asn92 and Asp114 interact with each other via hydrogen bonding (Fig. 5a). The enzyme remains in the closed conformation for several nanoseconds (Fig. 5b), which is sufficient time for the proton transfer reaction to occur^19^. During most of the simulation time, however, the loop is open (Fig. 5b), indicating that the closed conformation does not last long enough to obstruct the binding site.

### GH45 sequence analysis

To exploit the sequence space of the GH45 family we used the Peptide Pattern Recognition (PPR) bioinformatics method to analyze the GH45 primary structures and related sequences found in GenBank. This analysis divided the GH45-like sequences into 17 PPR groups (SMI). Out of these groups, seven contain sequences classified as subfamily A, three groups include sequences classified as subfamily B and two groups (group 3 and group 5) contain sequences classified as subfamily C (Table 1). Interestingly, Asn92 is conserved in the sequences of group 5, which includes *Pc*Cel45A, but not in the group 3 where only eight of the 47 sequences have Asn residue in the correspondent position and the most frequent residue is Ser (Table 1). Alignment of two of the highest scoring sequences in group 3 with two of the highest scoring sequences in group 5, including *Pc*Cel45A, shows that the sequence around residue 92 in *Pc*Cel45A is highly similar in the group 3 proteins although they predominantly have Ser residue in the position equivalent to Asn92 in the *Pc*Cel45A structure (Figure S8).

**Table 1:** Cross comparison of peptides in the PPR groups of the expanded GH45 family.

Group	1	2	3	4	5	6	7	8	9	10	11	12	13	14	15	16	17	SubFamily
**1**	**49**	9	0	0	0	15	5	0	6	13	0	0	0	0	0	1	0	**subA**
**2**	9	**70**	0	0	0	7	3	0	5	0	0	0	0	0	0	4	0	**subA**
**6**	15	7	0	0	0	**56**	4	0	5	2	0	0	0	0	0	1	0	**subA**
**7**	5	3	0	0	0	4	**70**	1	5	2	0	0	0	0	0	2	0	**subA**
**9**	6	5	0	0	0	5	5	0	**70**	0	0	0	0	0	0	0	0	**subA**
**10**	13	0	0	0	0	2	2	0	0	**70**	0	0	0	0	0	1	0	**subA**
**15**	0	0	0	2	0	0	0	1	0	0	0	0	0	0	**70**	0	0	**subA**
**16**	1	4	0	0	0	1	20	0	0	1	0	0	0	0	0	**70**	0	**subA**
**4**	0	0	0	**70**	0	0	0	1	0	0	1	0	0	1	2	0	0	**subB**
**11**	0	0	0	1	0	0	0	0	0	0	**70**	0	0	7	0	0	0	**subB**
**14**	0	0	0	1	0	0	0	0	0	0	7	0	0	**70**	0	0	0	**subB**
**3**	0	0	**70**	0	4	0	0	0	0	0	0	0	0	0	0	0	0	**subC**
**5**	0	0	4	0	**70**	0	0	0	0	0	0	0	0	0	0	0	0	**subC**
**12**	0	0	0	0	0	0	0	0	0	0	0	**70**	0	0	0	0	0	**swollenin**
**8**	0	0	0	1	0	0	1	**70**	0	0	0	0	0	0	1	0	0	—
**13**	0	0	0	0	0	0	0	0	0	0	0	0	**70**	0	0	0	0	—
**17**	0	0	0	0	0	0	0	0	0	0	0	0	0	0	0	0	**70**	—

The table show the number of conserved peptides shared between the groups. On the right column, the groups are categorized in subfamilies A, B or C, or as swollenins.

PPR sequence analysis of members of family GH45 is in congruence with division of GH45 into subfamily A, B and C with respect to that no PPR grouping includes members of more than one subfamily. Further, PPR-based analysis of presence of patterns of conserved peptides divides each subfamily into two or more PPR groups: GH45C is divided into two PPR groups (3 and 5); GH45B into three groups (4, 11, 14); and GH45A into 8 groups (1, 2, 6, 7, 9, 10, 15, 16). Such further division of subfamilies reflects differences in the evolutionary conserved peptide patterns.

## Discussion

Previous phylogenetic analysis demonstrated that *Pc*Cel45A is a representative member of the subfamily C from GH45 and is distantly related to canonical and well-characterized members of GH45^6^. Moreover, the analysis suggests the *Pc*Cel45A has higher structural similarity with expansins than with other members of the GH45 family. While Asp114 (relative to *Pc*Cel45A) is conserved in members both from subfamily A and C, the Asp10, which is described as a conserved proton acceptor in *Hi*EGV and *Ma*Cel45^7,8^, is absent in *Pc*Cel45A. Closer examination of *Pc*Cel45A structure bound to cellobiose revealed that the Asp114 side chain in the *Pc*Cel45A structure is inserted in a hydrophobic environment and its carboxyl group forms a hydrogen bond to the O4 atom of the +1 subsite glucopyranosyl unit. This suggests that Asp114 acts as a catalytic proton donor and protonates the glycosidic oxygen, as discussed in previous studies of *Hi*EGV^7,9^. In line with the structural observation, the mutation of Asp114 to alanine resulted in total loss of catalytic activity.

**Figure 6:**
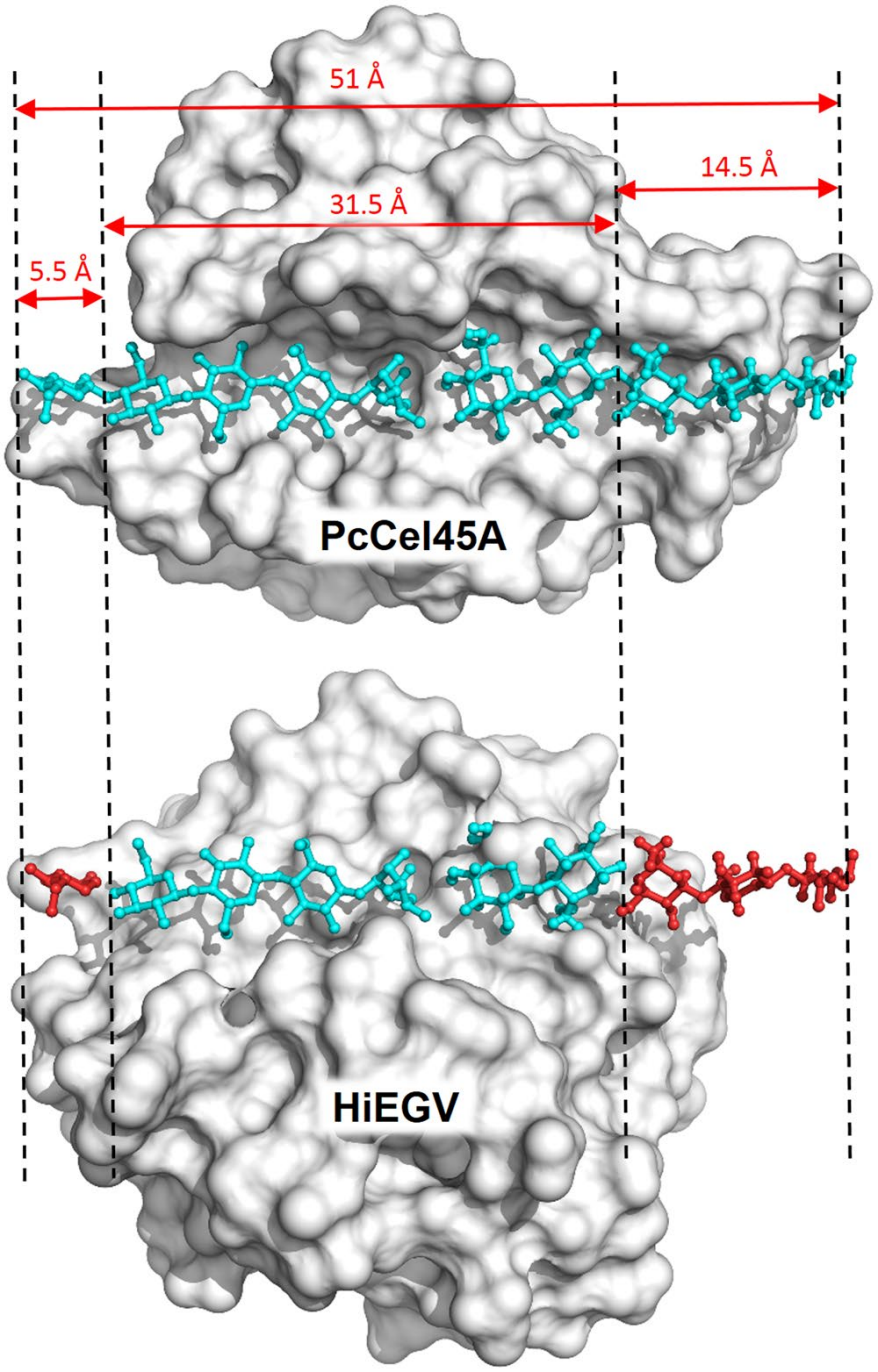
Difference in length of the active site in GH45. At the top, the surface view of *Pc*Cel45A with two cellopentaose molecules (light blue) fitting tightly along the whole length of the positive and negative subsites of the active site, respectively. Below, *Hi*EGV (PDB 1HD5) has a considerably shorter active site, which is unable to accommodate substrates longer than cellobiose at the positive subsites.

While most EGs including other members from GH45 family^6–9^ release cellobiose as a final product^20^, *Pc*Cel45A preferentially produces C3-C7 products (Figs 1 and S3). When *Pc*Cel45A (cellopentaose complex, PDB id: 3 × 2 M) and *Hi*EGV are compared, one can observe that the former exhibits a much longer active site groove as compared to the canonical members of GH45 family (Fig. 6). This much wider binding site is consistent with the formation of cellotriose, cellotetraose and larger products rather than cellobiose which is one of the most important inhibitors of cellulases^21^. Simulations of the *Pc*Cel45A bound to two substrates C4 + C3 which mimic a hydrolyzed C7 chain demonstrate that while C4 (bound to the negative subsites) remained tightly docked to the enzyme during the whole simulation lasting 150 ns, the C3 (bound to the positive subsites) dissociated in 10–30 ns, indicating that the substrate interacts more strongly with the negative than with the positive subsites (Figure S6A). In addition, simulations of the *Pc*Cel45A bound to the cellobiose (C2) molecule present in our crystallographic structure and occupying the +1 and +2 subsites, showed that it dissociates in a shorter time scale than C3 (Figure S6B). This suggests that interactions with residue Tyr67 are important for retaining the product of enzymatic reaction.

We also simulated *Pc*Cel45A in complex with C6 (cellohexaose) bound to the −3 to +3 subsites (hence, interacting with Tyr67) and, alternatively, bound to the −4 to +2 subsites (interacting with Trp154). In both cases, the chain readily assumed a non-productive configuration due to conformational alterations at the positive subsites (Fig. 3), which indicates that both aromatic residues at the ends of the *Pc*Cel45A binding cleft are important to properly hold the substrate for hydrolysis. This interpretation was further corroborated by simulations of the mutants Y67A and W154A complexed with C7 where the substrate assumed non-productive conformations faster than in the WT *Pc*Cel45A (Fig. 3). Similar results were obtained for the mutants Y18A and D85A (Fig. 3) which exhibited much lower activity than the WT enzyme. The time during which the substrate is found in the productive conformation is shown in Fig. 3. In Figure S7, the time history of the RMSDs of the substrates is shown, revealing the unbinding events. Taken together, these results suggest that *Pc*Cel45A does not effectively bind oligosaccharides shorter than C7 and that the aromatic residues Tyr67 and Trp154, as well as residues Tyr18 and Asp85, are essential for productive binding. Since the aromatic residues stabilize longer saccharide chains, they provide a rationale for comprehending the profile of generated product observed for *Pc*Cel45A, in contrast to the classical GH45 subfamily A enzymes such as *Hi*EGV. Indeed, *Pc*Cel45A, a member of subfamily C, generates much longer oligosaccharides as compared to *Hi*EGV.

The *Pc*Cel45A structure is marked by the presence of aromatic residues such as Tyr18, Tyr67 and Trp154 within the active site. These residues are mostly likely involved in the stabilization of the substrate by hydrophobic interactions. In fact, in our MD simulations, while wild-type enzyme remains bound to the substrate for up to 100 ns (which is probably sufficient to complete catalysis), cellohexaose detaches from the mutants Y18A and Y67A at the beginning of the simulations (Figure S7). This observation is in line with the faster migration pattern of mutants Y18A and W154A in the β-glucan matrix, presumably due to their weaker interactions with this substrate (Fig. 1).

Comparison of the active site shape for the GH45 subfamily A members *Ma*Cel45A and *Hi*EGV show that these enzymes lack aromatic residues at the extremities of the active site (comparable to Tyr67 and Trp154), but also that the structure exhibits large loops that can lock in and hold the substrate as exemplified by *Hi*Cel45^7^ (Fig. 2). On the other hand, *Pc*Cel45A has no large loops capable of embracing the substrate. The hydrophobic interaction provided by the aromatic residues mentioned above compensate in part for this lack of effective loop-substrate interactions. In this scenario it is likely that the substrate is retained by the enzyme for sufficient time to complete hydrolysis (Fig. 3a).

The loss of 50% of enzyme activity and the decrease of substrate affinity caused by the mutation in Trp154 (Fig. 1) can be understood by comparing the *Pc*Cel45A structure with that of expansins (Fig. 2 and Figure S9). The Trp154 amino acid residue is located in the active site groove of the *Pc*Cel45A structure and overlaps with the domain 2 of expansins, which indicates the possible importance of this residue for substrate binding and catalytic activity. The function of the domain 2 in expansins is not entirely known, but since this domain contains highly conserved aromatic residues, it has been hypothesized that domain 2 might be involved in a polysaccharide recognition and binding via a ring-stacking mechanism^16,22^. We speculate that since *Pc*Cel45A is a single domain enzyme and does not have a substrate binding module appended to it, the substrate recognition motif might be embedded in the catalytic domain itself in a way that resembles single domain cellulases, such as Cel12A, for example^23^. In the case of canonical GH45, the large loops can embrace and hold the substrate bound to the active site (Fig. 6). The substrate recognition mechanism of *Pc*Cel45A is likely to be mediated by the aromatic residues within the cleft, such as Tyr18, Tyr67, and Trp154 (Fig. 3).

After a hypothesis for the catalytic mechanism of *Pc*Cel45A, which involves crucial participation of the imidic form of Asn92 has been proposed^10^, we applied the PPR technique to verify if this catalytic mechanism could be universal for subfamily C. Therefore we classified the enzyme sequences annotated as GH45 members in the CAZy database in groups and observed common patterns among the sequences. Two distinct groups were readily identified in PPR analysis as containing GH45 subgroup C sequences (groups 3 and 5). Interestingly, only the group 5, to which *Pc*Cel45A belongs, has a conserved Asn in the position 92. This result clearly indicates that the recently proposed “Newton’s cradle” proton relay catalytic mechanism, involving Asn92 cannot be universal, nor can it be the only catalytic mechanism utilized by all members of GH45 subfamily C. Most of the enzymes that belong to group 3 do not have essential Asn92 but instead have a Ser residue in this position. Therefore at least one additional catalytic mechanism remains undescribed for GH45 enzymes of the subfamily C in cases when both Asp10 and Asn92 are missing. It is also interesting to note that when the peptides generated in the PPR analysis are mapped at the level of enzyme structure, a peptide flanking Asn92 is observed as a unique peptide for the PPR group 5 sequences (Figure S8), which again indicates that this region is a hot spot for this specific group of enzymes. Notably, the specific patterns of conserved peptides for each of the PPR GH45 groups can be used to discover more of such enzymes. For example, by analyzing metagenomes of habitats with different ecological, metabolic or physiological specialization, a new conceptual understanding of possible structural and functional differences between different groupings may hereby be gained.

**Figure 7:**
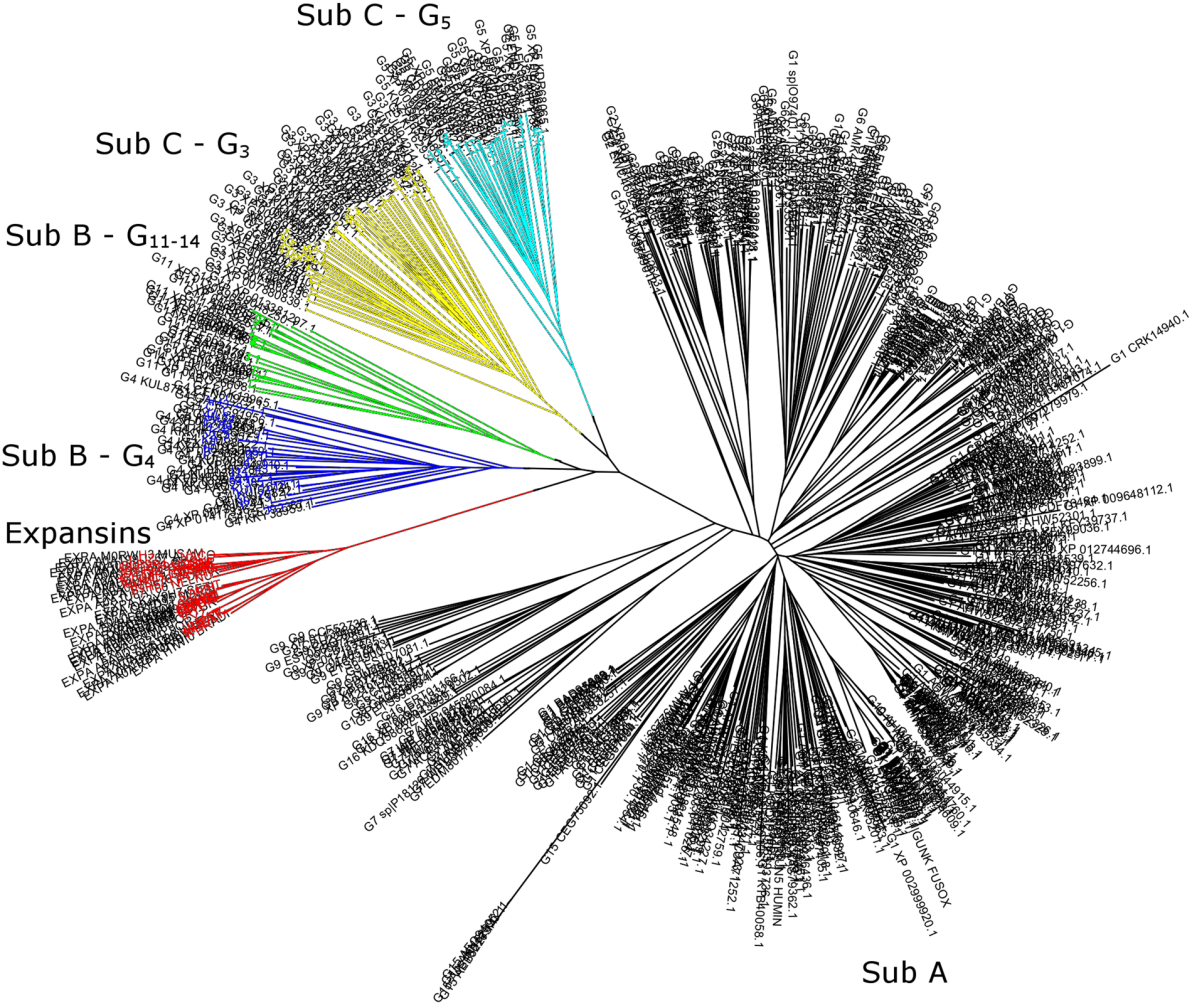
Phylogenetic analysis of GH45 and expansin sequences. The GH45 sequences used in PPR analysis were aligned together with 30 expansin sequences retrieved from a search against the referenced proteome database using jackhmmer (http://hmmer.org). The sequences were aligned with Clustal Omega and the phylogenetic tree was analyzed with MEGA7. The branch containing the expansin sequences is shown in red. The branches containing GH45 enzymes from subfamily B are shown in blue and green. The sub-branch shown in blue contains sequences grouped in group 4 by PPR, while the sub-branch shown in green contains sequences from groups 11 and 14. The branches shown in yellow and cyan contain GH45 enzymes from subfamily C, from groups 3 and 5, respectively, in PPR analysis. The largest branch (shown in black) contains sequences from GH45 subfamily A. Notably, the division of this branch in sub-branches is also correlated with the grouping observed in PPR analysis.

Finally, what evolutionary relationships would be expected for GH45 enzymes and expansins? Our structural comparison suggests that GH45 enzymes from subfamilies B and C are closely related to each other and also have higher structural similarity to expansins than to GH45 enzymes from subfamily A. On the other hand, expansins lost most of their hydrolytic activity, which is preserved for all members of GH45 family. These observations suggest that GH45 enzymes and expansins may share a common, not too evolutionary distant, ancestral gene. In fact, a phylogenetic analysis of the GH45 enzymes used in PPR analysis and 30 additional expansin sequences revealed that expansins form a branch in the phylogenetic tree that is closer to the branches where subfamilies B and C are situated than to the branch where subfamily A enzymes are grouped. Additionally, within the same subfamily branch, the enzyme sequences are clustered in groups with a distribution that is very similar to the group pattern observed in PPR analysis (Fig. 7), suggesting that PPR grouping has a clear correlation with the phylogenetic distribution of the sequences used in the analysis. Taken together, the sequence analyses suggest that within the GH45 subfamily C, a subgroup of endoglucanases (members of group 3 in PPR analysis) do not have a conserved Asn92 (PcCel45A numbering) to act as a general base, thus indicating that a new catalytic mechanism has to be employed by these enzymes. Additional investigations make themselves necessary to fully uncover the catalytic mechanisms of the enigmatic GH45 subfamily C of enzymes.

## Methods

### Cloning procedure

Initial nineteen residues (MAKLSMFLGFVAVATLASA) were predicted as a signal peptide and were removed from the cloned sequence^24^. The gene encoding *Pc*Cel45A was amplified from a cDNA library and cloned into pEXPYR + vector^25^ using a ligation-independent cloning protocol^26^. Details about protocol, primers and vectors are given in SMI and Table S1.

### Site-directed mutagenesis

To investigate the function of individual residues, we prepared a set of mutants based on interactions between *Pc*Cel45A and cellobiose. Residues Tyr18, Tyr67, Asp85, Asp114 and Trp154 were mutated to alanines using a site-directed mutagenesis technique and primers were designed with HTP-OligoDesigner^27^. Procedures were based on an inverse polymerase chain reaction (PCR) method^28^ in which the entire plasmid DNA is amplified by PCR. Details of the protocol, primers and vectors are given in SMI and Table S1. The mutant Y65A did not yield any soluble enzyme and for that reason was not characterized.

### Heterologous expression and purification of PcCel45A

The *Aspergillus nidulans* strain A773, kindly provided by Prof. Rolf Prade (OSU, USA), was used for heterologous expression^25^. The transformation procedure was performed according to Tilburn^29^. Positive transformants were selected in a small test using Congo red-stained agar to detect cellulase activity^30^. Protein expression was carried out using 6 L of minimal medium with 3% maltose as inducer and incubation under static conditions at 37 °C for 60 hours^25^. Cells were harvested and the supernatant was concentrated with tangencial flow filtration. The supernatant containing secreted *Pc*Cel45A was injected in a Q-sepharose (GE Healthcare, UK) column equilibrated with Buffer A (20 mM Tris-HCl, pH 8.5), and eluted with Buffer A increased with 1.0 M NaCl. Purity of the samples was confirmed by a SDS-PAGE analysis on 15% polyacrylamide gels (Figure S1). Mutants were purified similarly to native *Pc*Cel45A.

### Enzymatic activity

Enzymatic activity of *Pc*Cel45A and its mutants was evaluated colorimetrically using CMC as a substrate and dinitrosalicylic acid (DNS) as a chemical agent to reveal the quantity of reducing sugars generated by enzyme activity^31,32^, as described in SMI.

### Hydrolysis product analysis by high performance anion exchange chromatography (HPAEC)

Hydrolyzed products of PASC were analyzed by HPAEC. The hydrolyzed products were separated from insoluble PASC and analyzed using a DIONEX ICS3000 instrument equipped with the CarboPac PA1 4x250 mm column (DIONEX). The column was pre-equilibrated with 100 mM NaOH for 5 minutes at a flux of 1 mL.min^−1^. The saccharides were then resolved using a gradient from 100 mM NaOH/0 mM C_2_H_3_NaO_2_ to 100 mM NaOH/150 mM C_2_H_3_NaO_2_ over 20 minutes followed by washing step in 100 mM NaOH/1 M C_2_H_3_NaO_2_ for 2 minutes and equilibration with 100 mM NaOH for 5 minutes.

### Soluble polysaccharide binding assays

The capacity of *Pc*Cel45A and its mutants to bind to β-glucan was determined by affinity gel electrophoresis, as described by Duan *et al*.^33^. Briefly, 0.5% β-glucan was added to the polyacrylamide matrix, and results were compared to the mobility of a wild-type enzyme. Electrophoresis was conducted at 100 V and 4 °C, over a period of 4 h.

### Crystallization, data collection and structure solution

The protein was crystallized by the vapor diffusion method, at a protein concentration of 14.5 mg.mL^−1^. The crystals of *Pc*Cel45A were obtained in 0.5 M ammonium sulfate, 0.1 M Hepes pH 7.5 and 30% v/v (+/−) 2-methyl-2,4-pentanediol. The PcCel45A structure was solved by molecular replacement^34^, using the endoglucanase from *Mytilus edulis* (MeCel45A, PDB id: 1WC2, to be published) as a search model, which was identified by HHPred^35^. Details of data collection, structure solution, and refinement are given in SMI, while statistics from processing/refinement are given in Table S2. Analysis of the structures were performed with PyMOL, Dali server^15^ and PDBsum^36^.

### Molecular Dynamics simulations

The MD simulations were performed using the program NAMD^37^ with the CHARMM force field^38,39^ and TIP3P^40^ water model. Temperature and pressure were kept constant at 300 K and 1.0 atm under the Langevin thermostat and piston, respectively. Long-range interactions were handled with particle mesh Ewald (PME)^41^ and short-range interactions were truncated at a cutoff radius of 12 Å. Chemical bonds involving hydrogen atoms were constrained at their equilibrium lengths and a timestep of 2 fs was used to integrate the equations of motion. Analyses were performed with VMD^42^ and in-house codes. More details are given in SMI.

### Sequence Analysis

Amino acid sequences of all 340 GH45 proteins in the CAZy database^5^ were downloaded from GenBank. The sequences were classified with Peptide Pattern Recognition (PPR) as previously described^43^, using the parameters peptide length equal to 6, ten conserved peptides per protein and a number of conserved peptides per group equals 70^43^ to divide them into six groups. To expand the GH45 protein family, the top hit in each of the six PPR-generated GH45 groups and a selection of the unclassified sequences were used for BLAST search^44^ in GenBank. The 1000 top hits for each search were pooled and duplicates were removed. Next, protein domains in the sequences were mapped with CDD^45^ and domains were deleted that were clearly not related to GH45 and did not overlap with a GH45 domain. All sequences shorter than 51 amino acids after deletion of unrelated domains were removed. The 2975 curated protein sequences were grouped by PPR, using the default PPR methodology, described elsewehere^43,46^, and the proteins in each PPR group were analyzed with CDD. Finally, groups containing sequences not related to GH45 were removed. It is worth mentioning that the expansin sequences, being too distant, were not included in the curated sequence data. Sequence alignment and phylogenetic analysis were done with Clustal Omega^47^ and MEGA7^48^. For the phylogenetic analysis, expansin sequences were retrieved using Oryza sativa expansin1 (OsEXPA1) sequence as a query sequence in jackhmmer search (http://hmmer.org) against the referenced proteome database with a e-value cutoff of 1E-130. The sequences retrieved were manually curated to ensure that they had a typical expansin domain and the top 30 scored sequences were used for MSA and phylogenetic analysis in Clustal Omega and MEGA7.

### Data availability

Models of *Pc*Cel45A and *Pc*Cel45A-cellobiose complexes are deposited with PDB under the codes 5KJO and 5KJQ, respectively. The PDB accession codes 2ENG, 1O49, 3X2L, 2HCZ, 3D30, 4ENG, 3X2M, 1WC2 and GenBank entry BAG68300 were used in this study. Sequences from CAZy database were also used. All other data are available from the corresponding author upon reasonable request.

## Electronic supplementary material

Experimental procedures, Tables and Figures
