# Supplementary material for: Structure, computational and biochemical analysis of PcCel45A endoglucanase from Phanerochaete chrysosporium and catalytic mechanisms of GH45 subfamily C members
Source: Sci Rep. 2018 Feb 27;8:3678. doi: 10.1038/s41598-018-21798-9 (PMC5829257; doi:10.1038/s41598-018-21798-9)
Supplement: Supplementary file 1 — Experimental procedures, Tables and Figures [file 41598_2018_21798_MOESM1_ESM.pdf]

SUPLEMENTARY MATERIAL INFORMATION

Structure, computational and biochemical analysis of *Pc*Cel45A endoglucanase from *Phanerochaete chrysosporium* and catalytic mechanisms of GH45 subfamily C members

Andre S. Godoy[a,#], Caroline S. Pereira[b,#] Marina Paglione Ramia[a], Rodrigo L. Silveira[b], Cesar M. Camilo[c], Marco A. Kadowaki[a], Lene Lange[d], Peter K. Busk[d], Alessandro S. Nascimento[a], Munir S. Skaf[b] and Igor Polikarpov[a,*]

[a] São Carlos Institute of Physics, University of São Paulo, São Carlos 13566-590 São Paulo, Brazil

[b] Institute of Chemistry, University of Campinas, Campinas 13084-862, São Paulo, Brazil

[c] Centro de Tecnologia Canavieira, Fazenda Santo Antonio, PO Box 162, 13400-970 Piracicaba, São Paulo, Brazil

[d] Department of Chemical and Biochemical Engineering, Technical University of Denmark, Søltofts Plads, Building 229, 2800 Kgs. Lyngby, Denmark.

To whom correspondence should be addressed: Prof. Igor Polikarpov, São Carlos Institute of Physics, University of São Paulo, São Carlos, 13566-590 São Paulo, Brazil. Telephone: +55163373-8088. Fax: +55163373-9881; E-mail: ipolikarpov@ifsc.usp.br

Keywords: Endoglucanase; GH45 family; *Phanerochaete chrysosporium*; expansin; *Pc*Cel45A

Experimental Procedures

Cloning procedure. *P. chrysosporium* K-3 was cultivated in submerse fermentation in minimum liquid media containing glucose as sole carbon source. Total RNA was extracted with Trizol (Life Technologies, USA, CA) and used for first strand cDNA synthesis with "First Strand cDNA Synthesis" kit (Fermentas, USA, MA). The gene was amplified from cDNA by PCR, according to Phusion® High-fidelity DNA polymerase (New England Biolabs, USA, MA) specifications.

Site-directed mutagenesis. Expression vector containing the gene of interest is used as template for a PCR reaction. Forward (F) and reverse (R) primers anneal back to back to the plasmid which is entirely amplified by a proofreading polymerase that produces consistently blunt-ended DNA, as Phusion High-Fidelity DNA Polymerase (Thermo-Scientific, USA). After amplification, the PCR product is circularized by blunt-end ligation with T4 DNA ligase and, for this reason, primers must be synthesized with 5´end phosphorylation or PCR product must be phosphorylated by T4 Polynucleotide Kinase. Circularized PCR product is then transformed into *E. coli* DH10B competent cells, propagated and transformed as the native enzyme.

Enzymatic activity. The reaction containing 0.05 mL substrate (1% w/v) was carried out at 50 ºC in the universal buffer: 50 mM citrate/phosphate/borate buffer (pH range 2-10) for 15 minutes [1]. To stop the reaction, 0.1 mL of DNS was added and the solution was immediately heated at 95 ºC.[1] The release of reducing sugars was measured at 540 nm and compared with a glucose standard. The same procedure was used to find the optimal temperature for the enzymatic activity. The reaction was performed at pH 4.0, changing the temperature between 30 ºC and 80 ºC. To test substrate specificity, we used a set of different substrates. Recombinant PcCel45A (0.1 $mg.mL^{-1}$) was incubated with 1% (w/v) solutions of lichenan, β-glucan, galactomannan, CMC, chitin and chitosan, in a final volume of 60 µL containing 50 mM of sodium citrate pH 4.0 buffer, for 15 minutes, at 50 ºC. Mutants were tested similarly against CMC and β-glucan. For native enzyme, the Michaelis-Menten constant K_m, and the maximum velocity (V_{max}) were determined with OriginPro 8.0 (OriginLab, USA). All assays were performed in triplicate.

Thin Layer Chromatography (TLC). 0.5% PASC was incubated with 3.0 µM PcCel45A in 50 mM sodium citrate buffer (pH 4.0) at 50 ^{o}C for 24 h under constant agitation (1000 rpm). 200 µL of this reaction were centrifuged at 13,000 rpm and supernatant was collected. Then, 20 µL were applied to a silica gel 60 TLC plate (Sigma), and the run was performed in a butanol, acetic acid and water mixture (2:1:1). After two runs, TLC plates were sprayed with sulfuric acid (5%) ethanol mixture (95%) and heated to 95 ^{o}C

Data collection, phasing and refinement. Native crystals were harvested and cryoprotected by adding 10% (v/v) ethylene glycol and collected in the National Synchrotron Light Source (NSLS, USA) during the RapiData course at the beam line X4C (equipped with a MAR 165 CCD detector). The diffraction data was integrated with XDS.[2] The structure of enzyme-cellobiose complex was determined from the crystals collected from the crystallization drops saturated with cellobiose. After 3 hours, soaked crystals were collected at a Bruker APEX DUO single-crystal

diffractometer system equipped with a KAPPA goniometer and APEX II CCD detector. An exposure time was 10 seconds per frame for data collection with a -20° detector offset angle. The diffraction data was integrated with SAINT and scaled with SADABS.[3] Statistics of the data collection and space group determination were obtained with XPREP.[3] Data collection statistics are given in Table S2

Phases were solved by molecular replacement, with a model founded by the pairwise comparison of available crystal structures[4]. The best search model was the endoglucanase from *Mytilus edulis* (MeCel45A, PDB ID: 1WC2, to be published), with shares only 20% amino acid sequence identity with PcCel45A. This solution was used for further steps of density modification with RESOLVE.[5] After several cycles, model converged to a solution that was used for automatic building with AutoBuild.[5] Model was refined with Coot[6] and phenix.refine,[7] and validated with MolProbity.[8] Details of processing and refinement are given in Table S2.

Molecular dynamics and docking. Molecular dynamics simulations were performed for PcCel45A (wild type and mutants) in the absence and in the presence of several substrates. Initial coordinates of the substrate-free PcCel45A and PcCel45A-C2 complex were taken from the crystallographic structure reported in this work, and the other PcCel45A-substrate complexes were obtained according to the procedure described below. After removal of heteroatoms present in the crystal structures, hydrogen atoms were added according to predictions of protonation states at pH=4.0 with H++.[9, 10] All the water molecules present in the crystal structure were kept, and the whole system was inserted in a box at least 16-Å-thick. Sodium and chloride ions were added at the concentration of 0.1 M, with excess counterions to render the system electrically neutral.

The PcCel45A-C7 complex was obtained by taking the two cellotrioses (C3) present in the *Humicolas insolens* Cel45A (PDB code: 4ENG)[11] after structural alignment with PcCel45A, and completing the central glycosyl unit with several cycles of energy minimization (described below). The mutants and complexes of PcCel45A with C6, C4+C3 and X7 were obtained from the equilibrated PcCel45A-C7. Complexes of PcCel45A with C6 and C4+C3 were obtained from the equilibrated PcCel45A-C7 complex by removing from C7 a glucosyl unit and the glycosidic bond between subsites -1 and +1, respectively. Two PcCel45A-C6 complexes were obtained by deleting from C7 the glycosyl residue bound to the -4 and to the +3 subsite. The PcCel45A-X7 complex was obtained from the equilibrated PcCel45A-C7 complex by removing the hydroxymethyl groups from C7. The mutations Tyr25Ala, Tyr74Ala, Asp92Ala, Asn99Asp and Trp161Ala were performed with the psfgen module of NAMD (Phillips, 2005) and coordinates of the equilibrated PcCerl45A-C7 complex were used.

After taking the two C3 molecules from the *Humicolas insolens* Cel45A,[11] 2000 steps of energy minimization using the conjugated gradients (CG) algorithm were followed by 50 ps of MD keeping all the heavy atoms of the protein fixed. After that, a glucosyl unit was inserted in the space between the two C3 molecules and the following steps were conducted with a harmonic restraint between the catalytic acid Asp121 and the glycosidic oxygen atom that would be protonated during the catalysis: (1) 1000 steps of CG + 25 ps of MD with the protein the glycosyl units bound to subsites -4 to -2 and +1 to +3 fixed; (2) the same as step (1), but with only the glycosyl units bound to subsites -4, -3, +2 and +3 fixed; (3) same as before, but with glycosyl units bound to subsites -4 and +3 fixed; (4) same as before, but with all C7 chain free; (5) 1000 steps of CG + 50 ps of MD with only the alpha carbons fixed; (6) 50 ns of MD, keeping only the restraint between Asp121 and the glycosidic oxygen. After these preparation steps, which allowed the modeled C7 to relax in the PcCel45A, the PcCel45A-C7 complex was simulated until the C7 chain

assumed a non-productive binding mode. From the equilibrated PcCel45A-C7 complex, the other systems studied in this work were obtained. The mutants were further relaxed by the following procedure before the production runs: (1) 1000 steps of CG + 50 ps of MD with the protein fixed; (2) same as before, but with only the alpha carbons fixed; (3) 10 ns of restraint-free MD. Steps (1) to (3) were performed with a harmonic restraint on Asp121-glycosidic oxygen distance. Three independent simulations were conducted for each system. Trajectories lasted from tens to hundreds of nanoseconds, according to how long the substrate remained in the productive binding mode.

Results

Quality of the models. After X-ray data collection, the data were integrated in orthorhombic point group, and systematic absences revealed that the space group was $P\ 2_1 2_1 2_1$, with the crystallographic cell parameters equal to 45.6 Å, 58.6 Å and 63.6 Å. The asymmetric unit content was estimated using the Matthews coefficient (V_m = 2.35 $Å^3.Da^{-1}$).[12] This indicated one molecule of PcCel45A in the asymmetric unit cell (ASU), with 28% of solvent content. The X-ray structure of the apo-PcCel45A was refined, to a final R_{work} value of 15.11% and R_{free} = 17.83% at 1.46 Å resolution. The model contained 180 protein residues, 230 water molecules and 16 molecules of ethylene glycol. After refinement, 96.67% of the residues were attributed to the favored areas of the Ramachandran plot. The PcCel45A structure has two cysteine residues (64 and 94) with bad angles (SG-SG), possibly due to the radiation damage. PcCel45A - cellobiose complex was refined to 1.7 Å, and has a similar quality of apo-PcCel45A structure. Both structures are complete from residues 8 through 187. Despite being expressed by the fungal host *A. nidulans*, no post-translational modification was identified in the structure of the recombinant enzyme. The data collection and refinement statistics are given in Table S2.

Supplemental References

1. Miller, G. L. Use of Dinitrosalicylic Acid Reagent for Determination of Reducing Sugar, *Anal. Chem.* **31**, 426-428 (1959).
2. Kabsch, W. XDS, *Acta Crystallogr. D Biol. Crystallogr.* **66**, 125-132 (2010).
3. Bruker. *Saint*, SADABS and XPREP softwares, Bruker AXS Inc., Madison, Wisconsin, USA (2007).
4. Söding, J., Biegert, A., and Lupas, A. N. The HHpred interactive server for protein homology detection and structure prediction, *Nucleic Acids Res.* **33**, W244-W248 (2005).
5. Adams, P. D., *et al*. PHENIX: a comprehensive Python-based system for macromolecular structure solution, *Acta Crystallogr. D Biol. Crystallogr.* **66**, 213-221 (2010).
6. Emsley, P. and Cowtan, K. Coot: model-building tools for molecular graphics, *Acta Crystallogr. D Biol. Crystallogr.* **60**, 2126-2132 (2004).
7. Afonine, P. V. *et al*. Towards automated crystallographic structure refinement with phenix.refine, *Acta Crystallogr. D Biol. Crystallogr.* **68**, 352-367 (2012).
8. Chen, V. B. *et al*. MolProbity: all-atom structure validation for macromolecular crystallography, *Acta Crystallogr. D Biol. Crystallogr.* **66**, 12-21 (2010).
9. Gordon, J. C., Myers, J. B., Folta, T., Shoja, V., Heath, L. S., and Onufriev, A. H++: a server for estimating pKas and adding missing hydrogens to macromolecules, *Nucleic Acids Res.* **33**, W368-W371, (2005).

10. Myers, J., Grothaus, G., Narayanan, S., and Onufriev, A. A simple clustering algorithm can be accurate enough for use in calculations of pKs in macromolecules, *Proteins* **63,** 928-938, (2006).
11. Davies, G. J., Tolley, S. P., Henrissat, B., Hjort, C. & Schulein, M. Structures of oligosaccharide-bound forms of the endoglucanase V from *Humicola insolens* at 1.9 Å resolution. *Biochemistry* **34,** 16210–16220 (1995).
12. Matthews, B. W. Solvent content of protein crystals, *J. Mol. Biol.* **33**, 491-497 (1968).

SI Appendix, Tables

Table S1. Primers used for amplification of PcCel45A, pEXPYR and site-directed mutagenesis. Italic letters represent LIC tags.

	Primers
PcCel45A	Fw - *CAGGGCGCCATG*CTGACCGTCTCCGAGA
	Rv - *GACCCGACGCGGTTA*CGAAGGGGCAGTCCCC
pEXPYR+ vector	Fw - CAGGGCGCCATG
	Rv - GACCCGACGCGGTTA
Y18A	Fw - CACGATGGCTTCGGGCTG
	Rv - AAGGATGCCTGGCCAGTAGCC
D85A	Fw - GGTCACGGCCCTATGCCC
	Rv - TTTACAACGATCGTCTGCCCG
D114A	Fw - GTTCCACTTCGCTATCTGCGAGG
	Rv - GGCATACCGTGCTGGTTCG
W154A	Fw - GCCAGCTCGCGAACGGT
	Rv - CGCCGTCGGAGCCC

Table S2. Data collection and refinement statistics from PcCel45A native structure and it cellobiose complex. Values in parentheses refer to the highest resolution shell.

	**PcCel45A (PDB id 5KJO)**	**PcCel45A-cellobiose (PDB id 5KJQ)**
Data collection		
Space group	$P\ 2_1\ 2_1\ 2_1$	$P\ 2_1\ 2_1\ 2_1$
Cell dimensions		
a,b,c (Å)	45.6, 58.6, 63.6	45.3 58.15 62.9
α,β,γ (º)	90, 90, 90	90, 90, 90
Resolution (Å)	31.8 (1.47)	36.7 (1.7)
$R_{\mathrm{merge.}}$	4.3 (17.9)	11.3 (37.6)
I/σ*I*	12.5 (3.4)	8.4 (1.8)
Completeness (%)	97.3 (87.0)	99.0 (92.1)
Redundancy	7.4 (2.4)	5.71 (3.49)
Refinement		
Resolution (Å)	31.8 (1.47)	36.7 (1.7)
No. reflections	29354 (2568)	18567 (1694)
R_{work}	15.1 (24.5)	17.7 (23.7)
R_{free}	17.8 (24.9)	20.6 (27.6)
No. atoms (non-H)		
Protein residues	180	180
Ligands	16	28
Water	230	271
B-factors		
Protein	15.60	14.00
Ligands	29.30	22.40
Water	28.80	26.10
Root mean squared deviations		
Bond lengths (Å)	0.016	0.014
Bond angles (º)	1.52	1.23

SI Appendix, Figures

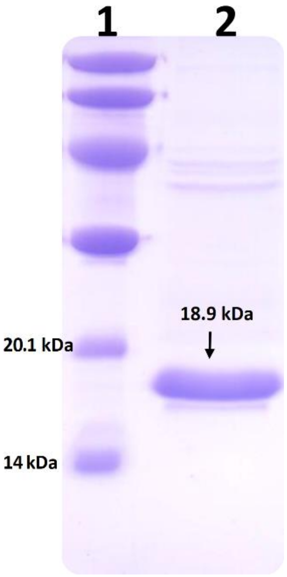

Fig. S1. SDS-Page 15% analysis of the purified recombinant *Pc*Cel45A: (1) Molecular markers, (2) purified recombinant PcCel45A.

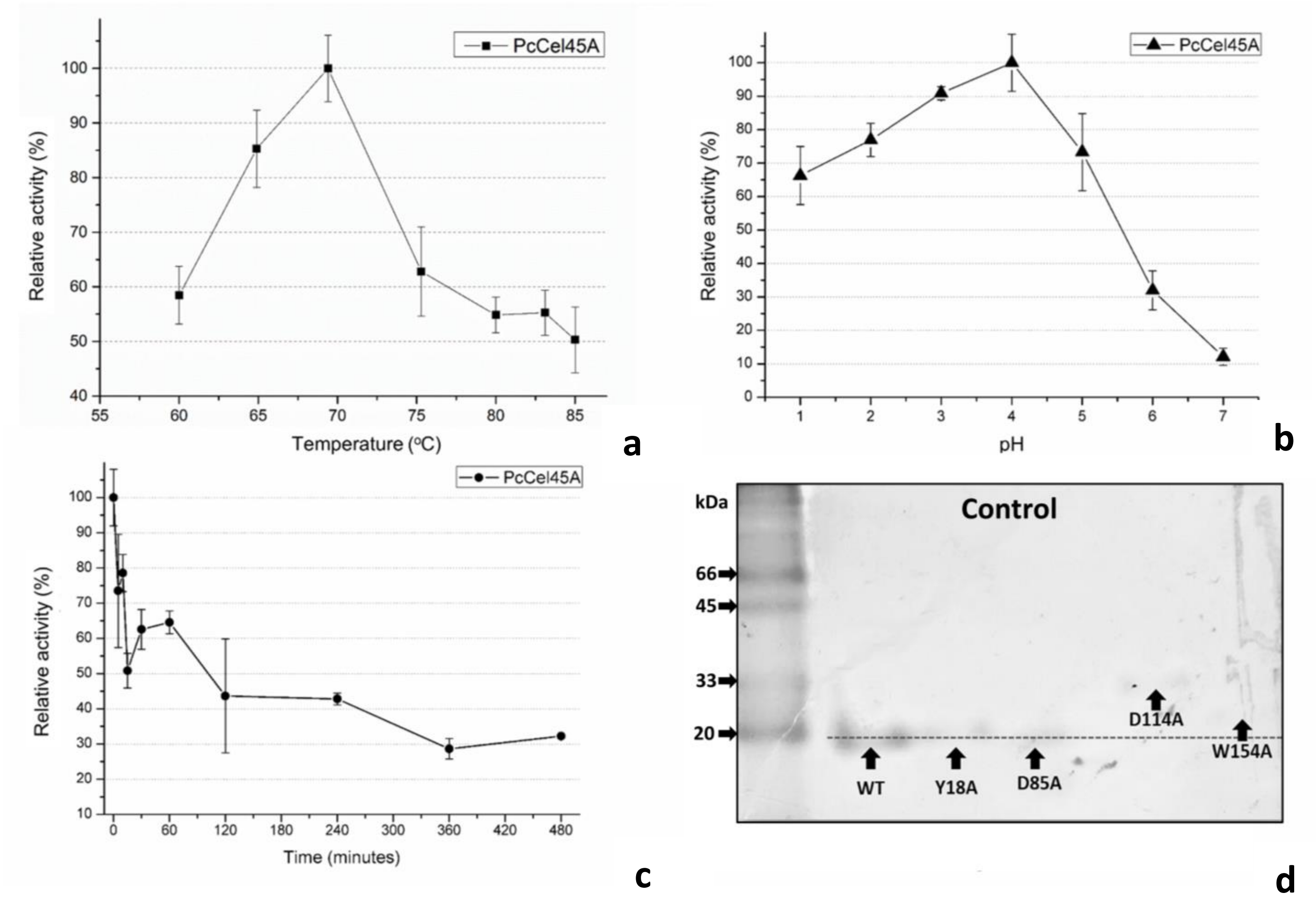

Fig. S2. Biochemical characterization of PcCel45A. (**a**) The relative activity of *Pc*Cel45A in different temperatures. (**b**) The relative activity of *Pc*Cel45A in different pH's. (**c**) The remaining activity of *Pc*Cel45A when incubated at 70 ^{o}C for different amounts of time. (**d**) Native electrophoresis for *Pc*Cel45A and its mutants.

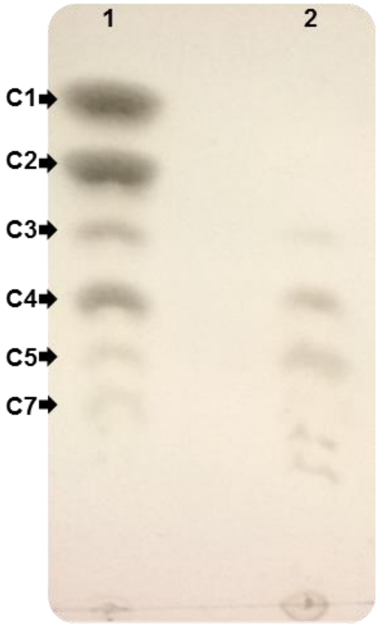

Fig S3. TLC analysis of reaction products generated by *Pc*Cel45A when incubated with 0.5% PASC. Lane 1: standard oligosaccharides; lane 2: reaction products.

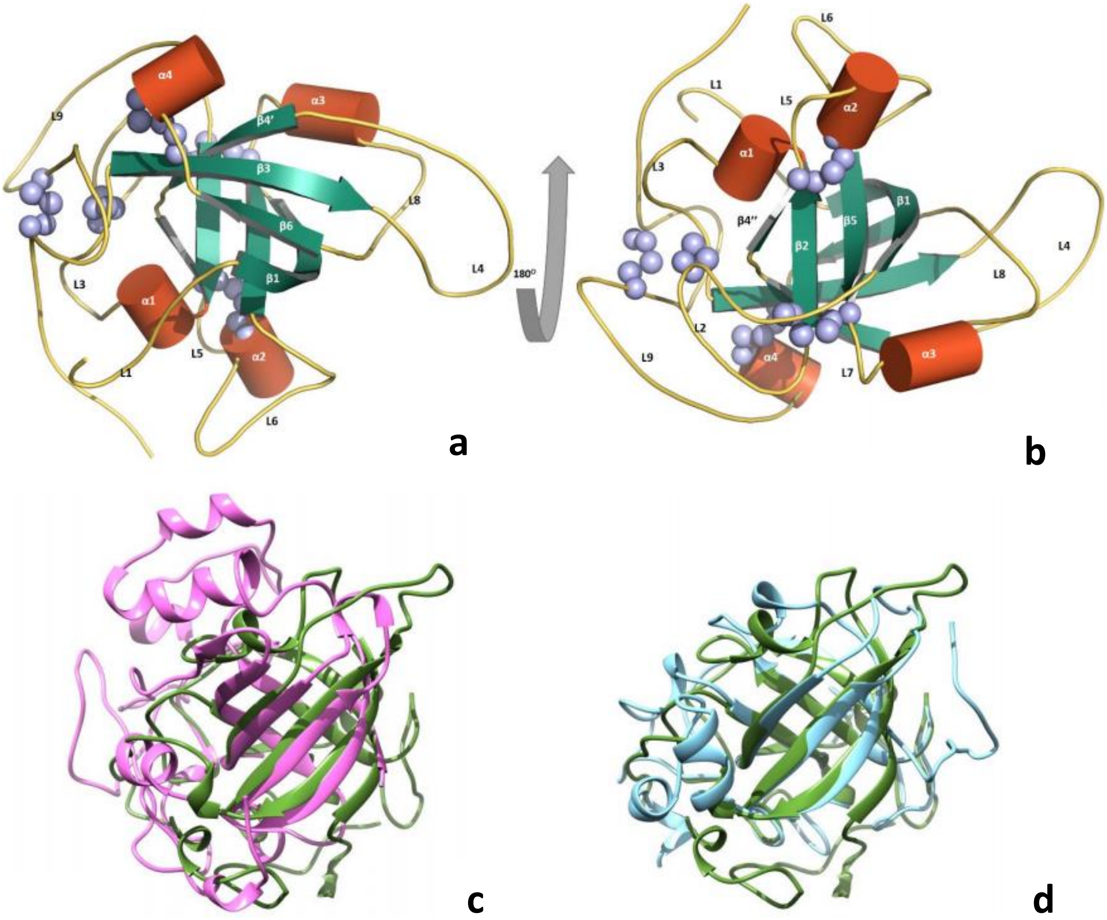

Fig. S4: Overall structure of PcCel45A. (**a** and **b**) The six-stranded β-barrel structure (β-sheets in green) followed by four α- helices (orange). Loops are presented in yellow. Cysteine residues involved disulphide bridges are shown as purple spheres. (**c**) Structural comparison between PcCel45A (shown in green) and the subfamily A representative HiEGV enzyme (PDBId 2ENG; in magenta). (**d**) Structural comparison between PcCel45A (in green) and the subfamily B representative MeCel45A (PDBId 1WC2, given in light blue).

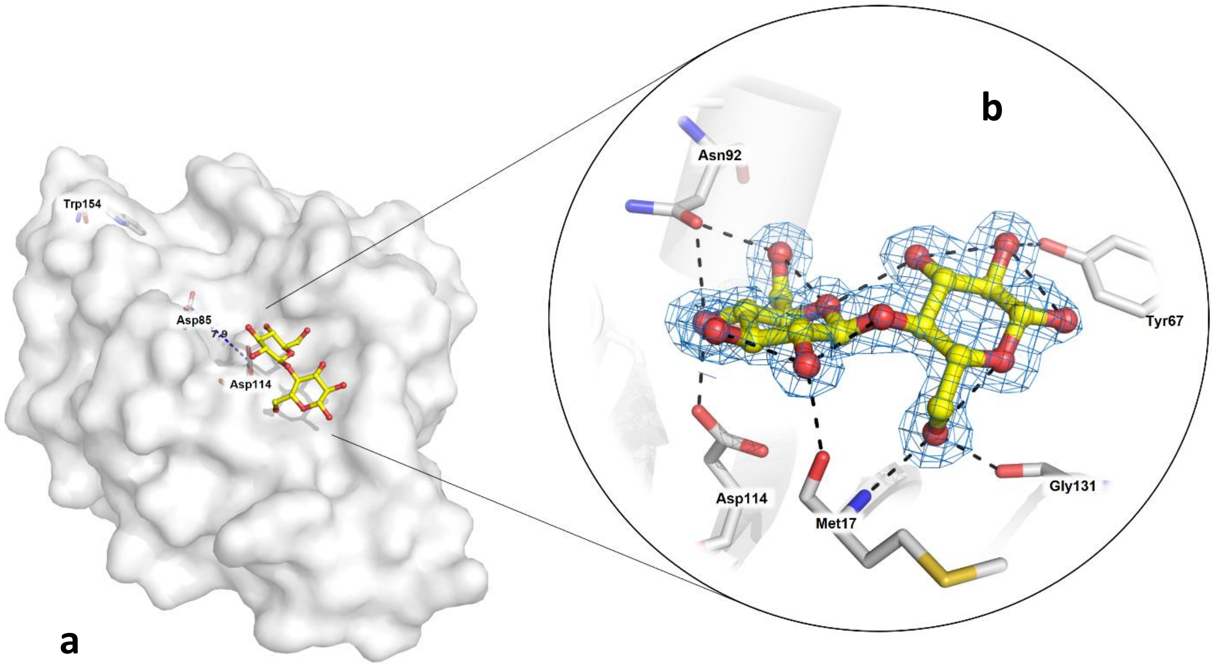

Fig. S5 Details of the cellobiose complex interaction with *Pc*Cel45A residues in the activite site. (**a)** The surface view of the groove and the positioning of cellobiose in the +1 and +2 sub-sites. Asp85 and Asp114, shown in sticks, are located at 8.0 Å from each other. (**b**) Closer view of the active site with depicted cellobiose and interacting residues. The 2Fo-Fc (1.0 sigma) map is depicted as a blue mesh.

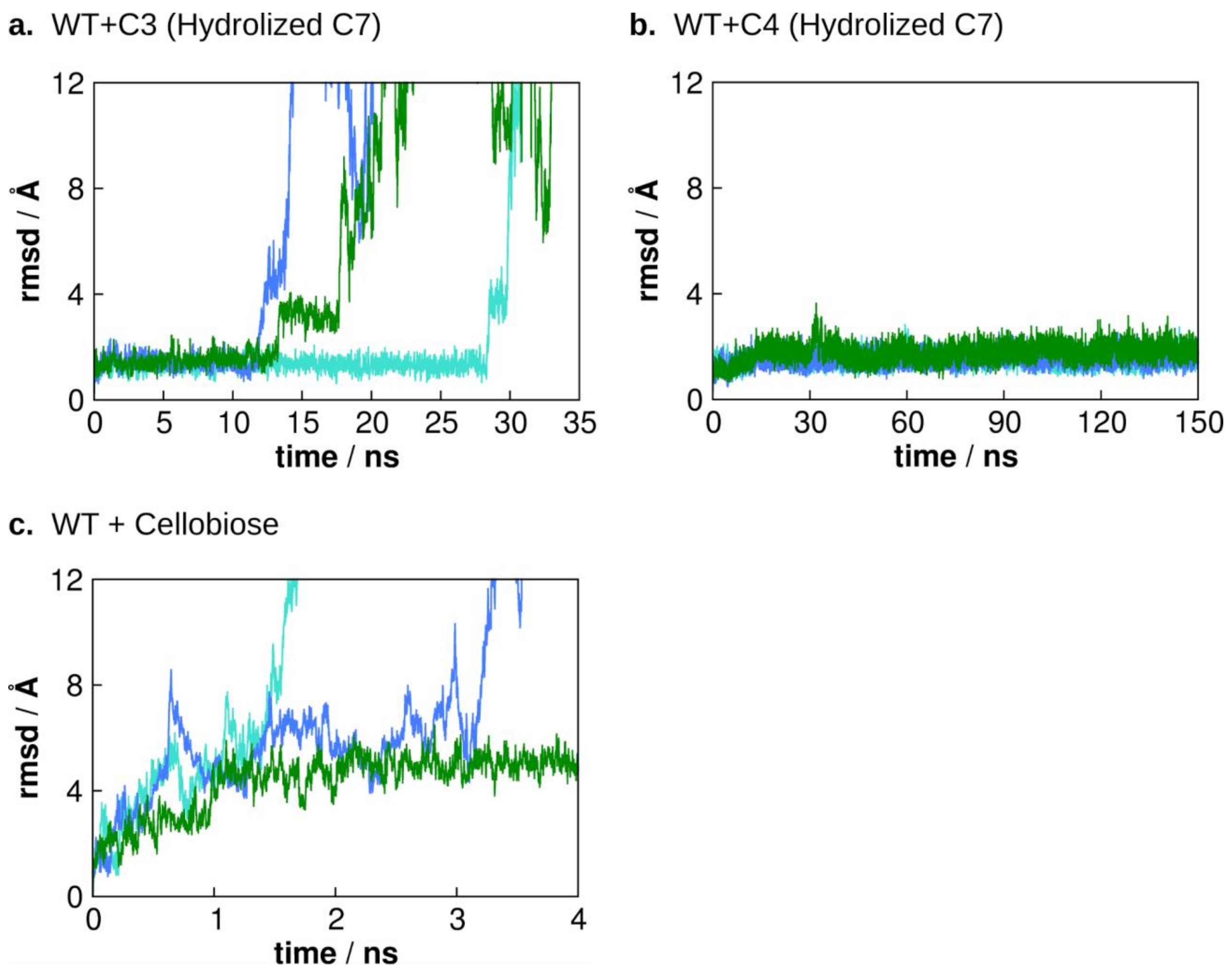

Fig. S6. The RMSD from different carbohydrates main chain bound to *Pc*Cel45A. Increases of the RMSD above ~2.5 Å indicate that the chain dissociates to the solution. Different colors represent different simulations of the same system.

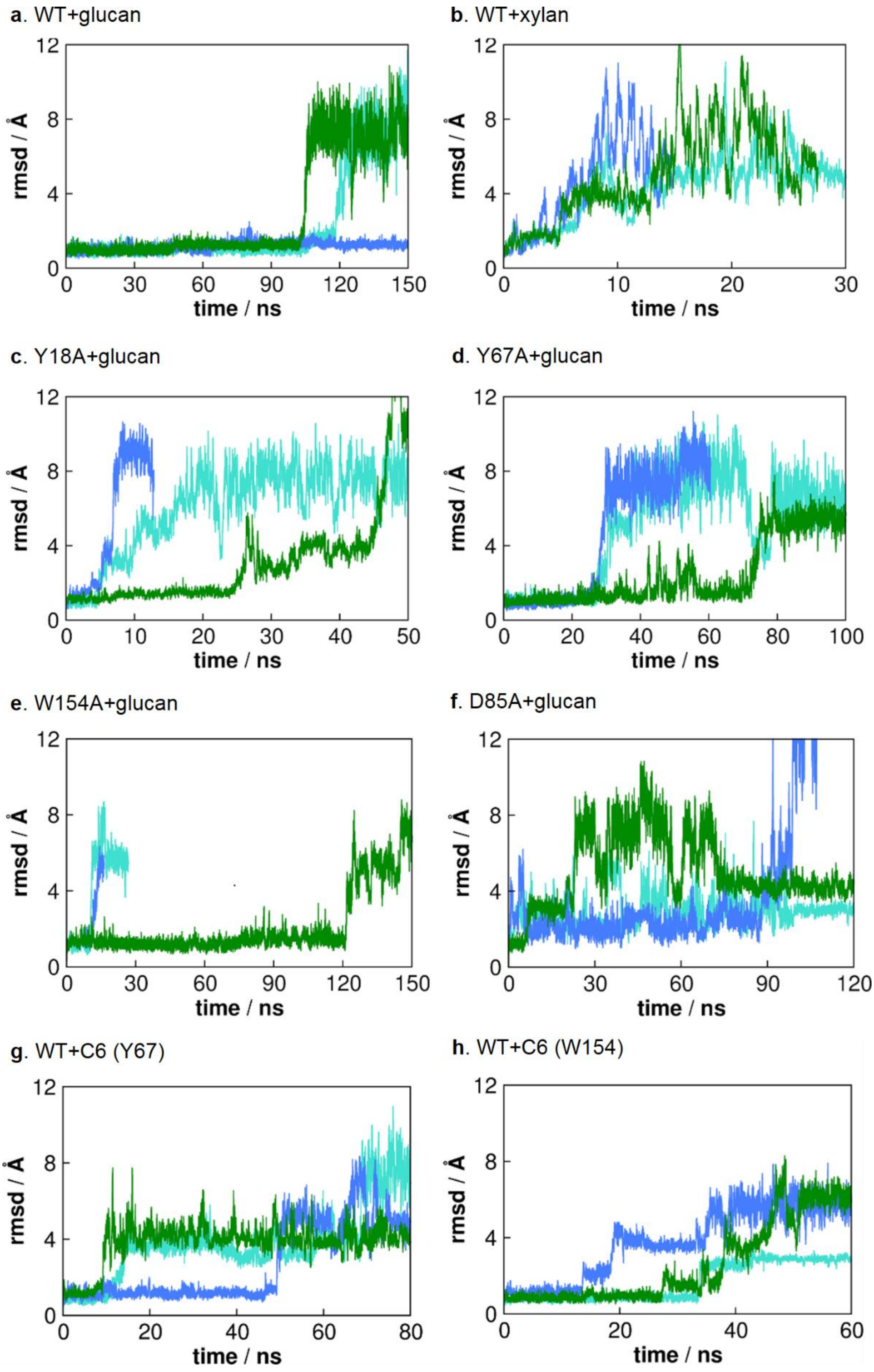

Fig. S7. The RMSD from different carbohydrates main chain bound to *Pc*Cel45A and mutants. When the RMSD fluctuates around ~2 Å, the C7 chain (glucan) is bound in the productive conformation. When the RMSD increases to ~4 Å, the C7 chain assumes a non-productive binding mode. Different colors represent different simulations of the same system.

a

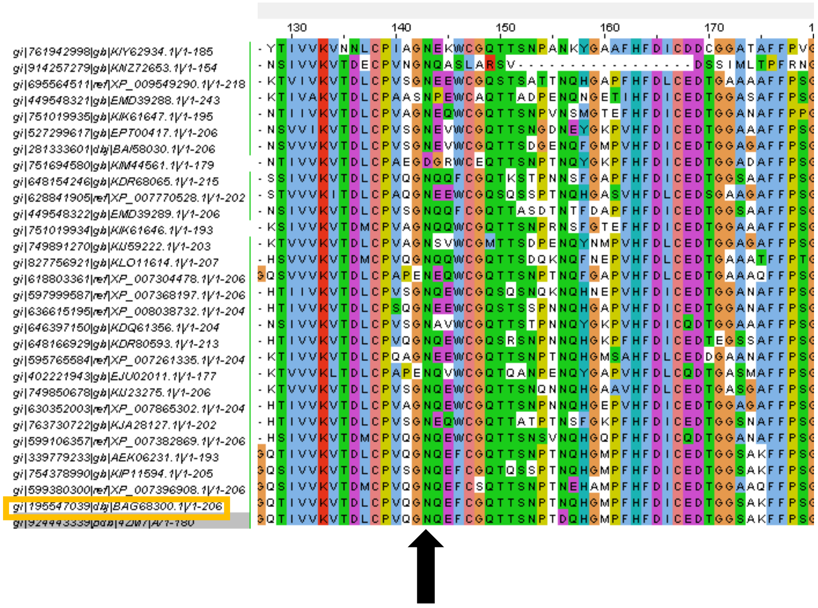

b

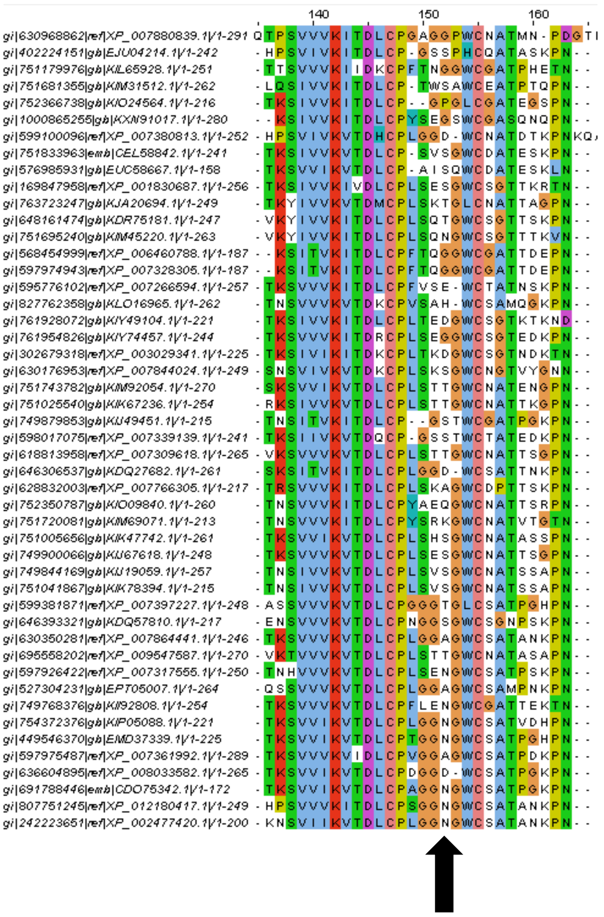

Fig S8. (**a**) Alignment of the sequences in PPR group 5. *Pc*Cel45A is indicated with an orange square and Asn92 is indicated with a blue arrow. (**b**) Alignment of the sequences in PPR group 3. The position corresponding to Asn92 in *Pc*Cel45A is indicated with a black arrow.

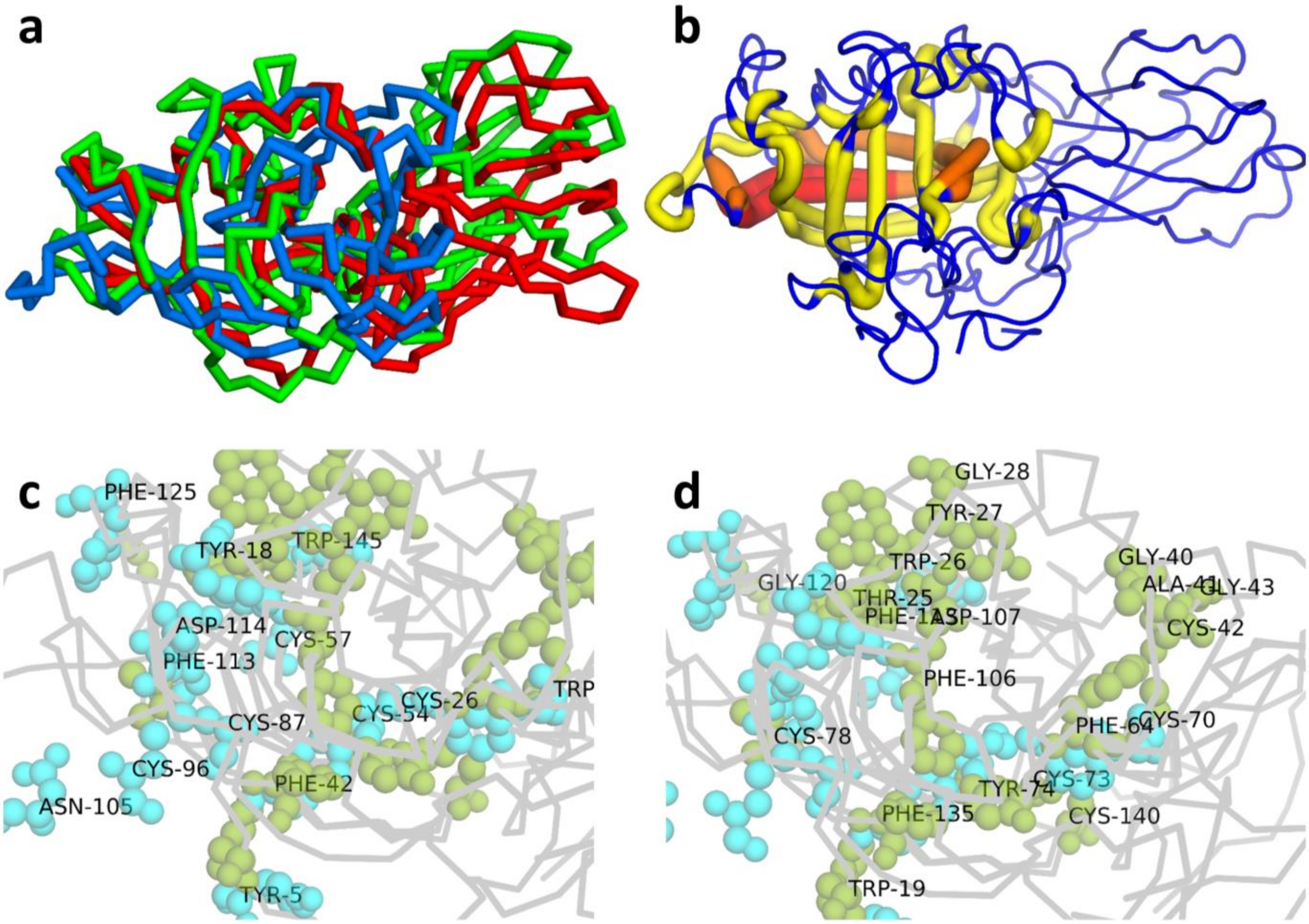

Fig. S9. Comparisons of *Pc*Cel45A, EXPB1 and EXLX1. (**a)** Ribbon superposition of PcCel45A (shown in blue), EXPB1 (in green) and EXLX1 (in red). **(b)** Alignment of PcCel45A and EXPB1 colored by conservation (blue is less conserved, while red is more conserved). **(c** and **d**) Conserved residues of PcCel45A (in blue, residues identification in **c)** and EXPB1 (in green, residues identification in **d)**
